# Fungal diversity driven by bark features affects phorophyte preference in epiphytic orchids from southern China

**DOI:** 10.1038/s41598-021-90877-1

**Published:** 2021-05-28

**Authors:** Lorenzo Pecoraro, Hanne N. Rasmussen, Sofia I. F. Gomes, Xiao Wang, Vincent S. F. T. Merckx, Lei Cai, Finn N. Rasmussen

**Affiliations:** 1 School of Pharmaceutical Science and Technology, Tianjin University, Tianjin, 300072 China; 2 Institute for Geoscience and Nature Management, University of Copenhagen, 1958 Frederiksberg C, Denmark; 3 Naturalis Biodiversity Center, 2332 AA Leiden, The Netherlands; 4 State Key Laboratory of Mycology, Institute of Microbiology, Chinese Academy of Sciences, Beijing, 100101 China; 5 Natural History Museum of Denmark, University of Copenhagen, 1350 Copenhagen K, Denmark

**Keywords:** Biodiversity, Community ecology, Ecosystem ecology, Forest ecology, Microbial ecology

## Abstract

Epiphytic orchids exhibit varying degrees of phorophyte tree specificity. We performed a pilot study to investigate why epiphytic orchids prefer or avoid certain trees. We selected two orchid species, *Panisea uniflora* and *Bulbophyllum odoratissimum* co-occurring in a forest habitat in southern China, where they showed a specific association with *Quercus yiwuensis* and *Pistacia weinmannifolia* trees, respectively. We analysed a number of environmental factors potentially influencing the relationship between orchids and trees. Difference in bark features, such as water holding capacity and pH were recorded between *Q. yiwuensis* and *P. weinmannifolia*, which could influence both orchid seed germination and fungal diversity on the two phorophytes. Morphological and molecular culture-based methods, combined with metabarcoding analyses, were used to assess fungal communities associated with studied orchids and trees. A total of 162 fungal species in 74 genera were isolated from bark samples. Only two genera, *Acremonium* and *Verticillium*, were shared by the two phorophyte species. Metabarcoding analysis confirmed the presence of significantly different fungal communities on the investigated tree and orchid species, with considerable similarity between each orchid species and its host tree, suggesting that the orchid-host tree association is influenced by the fungal communities of the host tree bark.

## Introduction

Epiphytism is one of the most common examples of commensalism occurring in terrestrial environments, which provides advantages, such as less competition and increased access to light, protection from terrestrial herbivores, and better flower exposure to pollinators and seed dispersal^1,2^. Vascular plant epiphytes represent a significant portion, up to one third, of tropical and subtropical plant species diversity^3^ and may contribute as much as 10% of the world’s total flora^4^. Roughly 60% of all epiphyte species are members of the Orchidaceae, the largest family in the plant kingdom, including approximately 20,000 species with epiphytic habit, in 500 genera^5–7^. With such a significant number of plants living as epiphytes, and such a high proportion of Orchidaceae representing the epiphytic lifestyle, phorophyte trees constitute crucial elements in many ecosystems, as they provide the suitable substrate and environmental conditions for a large variety of plants, and particularly for orchids to grow. However, although trees that carry epiphytic orchids play a fundamental role in orchid life cycle, little is known about this phorophyte-epiphyte relationship^8,9^. Epiphytic orchids exhibit varying degrees of phorophyte tree specificity. Some orchid species show a strong phorophyte preference, while other species associate with a broad range of tree hosts. For instance, Gowland et al. (2011)^10^ showed that three co-occurring orchid species belonging to Aeridinae, in Australia, were characterized by different level of phorophyte specificity, with *Sarchochilus parviflorus* showing a very narrow range of suitable host trees, *Sarchochilus hillii* being more generalist, and *Plectorrizha tridentata* showing an intermediate phorophyte specificity. Some studies suggest that epiphytic orchid distribution and abundance may be affected by phorophyte specificity^11,12^. However, our knowledge and understanding of factors that determine this specificity are still in their infancy^9^. Physical host tree traits such as bark stability, texture, and water absorbing capacity, age and trunk size, canopy structure, branching pattern and leaf size, may influence orchid seed germination as well as protocorm and seedling development, thus determining host suitability^13,14^. For instance, smooth and exfoliating barks may prevent attachment of seeds on a potential host^5,15^. Older trees, with greater diameter at breast height, provide larger area available for epiphytic orchid colonization^5,16^. Other important biophysical parameters of the epiphytic orchid germination niche can be microsite temperature, water availability, and humus presence^17,18^. Chemical factors may also be crucial to explain why a certain tree provides suitable substrate for some orchid species to establish and grow, while does not enable other species to colonize its bark^19^. Frei (1973)^20^ explained the absence of orchid epiphytes on some oak trees in Mexico with the presence of gallic and ellagic compounds in their bark that inhibited orchid seed germination. Among factors that may influence the orchid preference for a phorophyte, biotic elements certainly deserve to be considered. For instance, the presence of mosses on phorophyte bark may be critical for orchid germination success and establishment^21^. The presence of ants or termites may also determine the orchid colonization of a tree host^3,22^. Competing orchid seedlings that exploit the same carbon source, and vegetation that competes with orchids for water and nutrients and affects orchid light exposure, may reduce with their presence the establishment chances of an orchid species on a certain tree^9^. Finally, phorophyte bias could be related to the availability of mycorrhizal fungi that are essential for orchid seed germination and protocorm development^23–25^. Both presence and performance of suitable mycobionts on the barks of different trees, which may be affected by a number of microenvironment physico-chemical factors, could determine orchid epiphyte distribution in nature^9^. Besides, the presence of other non-mycorrhizal fungi, such as fungal parasites or antagonists that compete for the same nutrients, could influence the performance of suitable orchid mycobionts and therefore indirectly affect orchid distribution^26^.

We performed a pilot study to investigate why epiphytic orchids prefer or avoid certain phorophyte trees. This question becomes more puzzling when the preferred trees differ among epiphytes that seem to mutually exclude each other, within the same area. For this reason, we selected two orchid species, *Panisea uniflora* and *Bulbophyllum odoratissimum* co-occurring in a forest habitat in southern China. *P. uniflora* was found to grow on *Quercus yiwuensis* while not growing on *Pistacia weinmannifolia* trees. The latter phorophyte host carried *B. odoratissimum* that was never found to colonize *Q. yiwuensis*. We analysed a number of biotic and abiotic environmental factors potentially influencing the relationship between the studied orchids and trees, including bark and orchid root fungal diversity. We hypothesised: (i) that the tree host specialization of the studied orchids was influenced by the presence of specific orchid mycorrhizal fungi, which were in turn biased toward particular tree species, and (ii) that the specific features of the bark either directly affected the early establishment of the orchid seedlings or did so indirectly through the fungal community that was able to grow on the bark.

## Results

### Diversity of culturable bark fungi

A total of 258 fungal strains were isolated from the 36 bark samples collected from the three studied phorophyte species. These isolated strains belonged to 162 species in 74 genera mostly of Ascomycota, the great majority (66), and Basidiomycota (6), accounting for 93.4% and 5.8% of the fungal strain diversity, respectively. Only two strains, one isolated from *P. weinmannifolia* tree, identified as *Mortierella alpina* in the Mortierellaceae family, the other one from *Q. yiwuensis* tree, *Umbelopsis isabellina* (Umbelopsidaceae), represented Mucoromycota (Supplementary Table S1). Ascomycetous fungi belonging to *Cladosporium* (19 strains), *Cyphellophora* (11), *Fusicolla* (13), *Penicillium* (25), *Pestalotiopsis* (18), and *Trichoderma* (12) were dominant. Among them, the genus *Penicillium*, with the highest number of strains, also showed the highest species richness, with 10 isolated species, followed by *Pestalotiopsis*, which yielded 7 species, while *Cladosporium* and *Trichoderma* showed the third highest species diversity, both represented by 6 species. From the total *taxa* list, the most common species included *Fusicolla violacea* (5.6%), isolated from all the three phorophyte species, *Clonostachys rosea*, recorded on N- and B-trees (defined in “Methods” section below), and *Cyphellophora europaea*, only present on B-trees, both accounting for 3.9% of strains, *Penicillium wollemiicola* (3.4%), *Cladosporium cladosporioides* (3%), and *Pestalotiopsis microspora* (2.6%), while each of the 5 species *Cladosporium ramotenellum*, *Coniothyrium nitidae*, *Hypocrea lixii*, *Penicillium herquei*, and *Trichoderma harzianum* represented 1.7% of isolated strains. The highest fungal diversity was observed on neutral trees (N-trees, *B. percoriacea*) with 71 species in 49 genera (126 strains), followed by *P. weinmannifolia* (B-trees) with 47 (33 genera, 82 strains), and *Q. yiwuensis* (P-trees) with 31 (20 genera, 50 strains). Twenty-nine genera were exclusive of N-trees, 13 of B-trees, and 5 were only reported for P-trees. Nine genera (*Bionectria*, *Cladosporium*, *Clonostachys*, *Cylindrocladium*, *Fusarium*, *Fusicolla*, *Hypocrea*, *Penicillium*, *Pestalotiopsis* and *Trichoderma*) were in common for all the three tree species, 7 (*Alternaria*, *Annulohypoxylon*, *Biscogniauxia*, *Liberomyces*, *Massarina*, *Nectria* and *Xylaria*) were shared by N- and B-trees. Only two fungal genera *Letendraea* and *Talaromyces* were in common for N- and P-trees, while *Acremonium* and *Verticillium* were shared by P- and B-trees. Basidiomycetous fungi were mostly found to grow exclusively on N-trees, 5 out of the total 6 genera, including *Coprinellus* in the Psathyrellaceae family of Agaricales, *Deconica* (Strophariaceae, Agaricales), *Marasmius* (Marasmiaceae, Agaricales), *Marasmiellus* (Omphalotaceae, Agaricales), and *Peniophora* (Peniophoraceae, Russulales), with the exception of a single strain belonging to the genus *Lycoperdon* (Agaricaceae, Agaricales), which was isolated from the bark of B-tree (Supplementary Table S1). Bark fungi in vitro isolation attempts did not yield strains for a variety of basidiomycetes which were instead detected by metabarcoding analyses, such as *Rhizoctonia*-like fungi in Ceratobasidiaceae, Tulasnellaceae, and Sebacinales.

### Fungal diversity by Illumina sequencing

**Figure 1:**
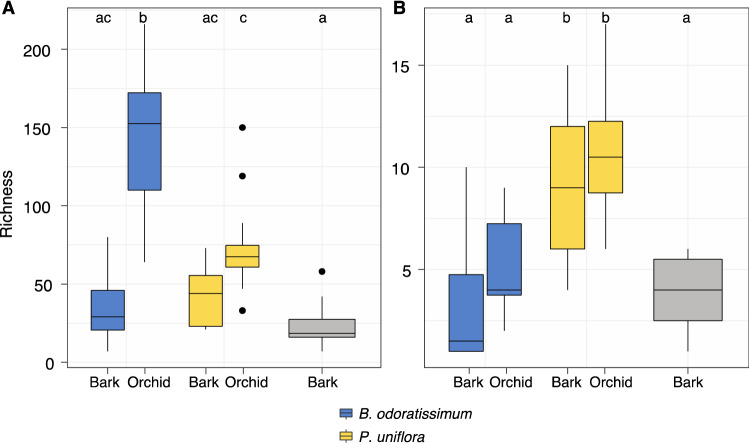
Richness of total (**A**) and orchid-associated (**B**) fungi associated with the orchid species *B. odoratissimum* and *P. uniflora*, and the bark of *P. weinmannifolia (blue), Q. yiwuensis* (yellow) and *B. percoriacea* (grey).

From the metabarcoding analysis performed on the total 60 samples collected from bark (36) and orchid roots (24), after all the filtering steps, we found 472 zOTUs (438,772 reads) in the total fungi dataset and 53 zOTUs (126,129 reads) in the orchid-associated fungi dataset. Total fungal richness was significantly different between orchids and bark (*F* = 58.46, *P* < 0.001), in which the orchid *B. odoratissimum* had significantly higher richness than the bark where it was collected (*P* < 0.001), but the orchid *P. uniflora* presented similar richness as the bark. There were no significant differences in richness between the fungal communities in the bark of the three tree species (Fig. 1A). Orchid-associated fungal richness was significantly different between orchids and bark (*F* = 5.64, *P* = 0.020) with no differences between orchids and the bark of their respective hosts trees. *P. uniflora* had significantly higher richness than *B. odoratissimum* (P = 0.027). Fungal communities in the bark of the tree that did not host any orchid (*B. percoriacea*) were significantly less rich than those in the orchid *P. uniflora* (P < 0.001) and its respective bark (P < 0.001), while no differences were found in relation to the orchid or bark of *B. odoratissimum* (Fig. 1B).

**Figure 2:**
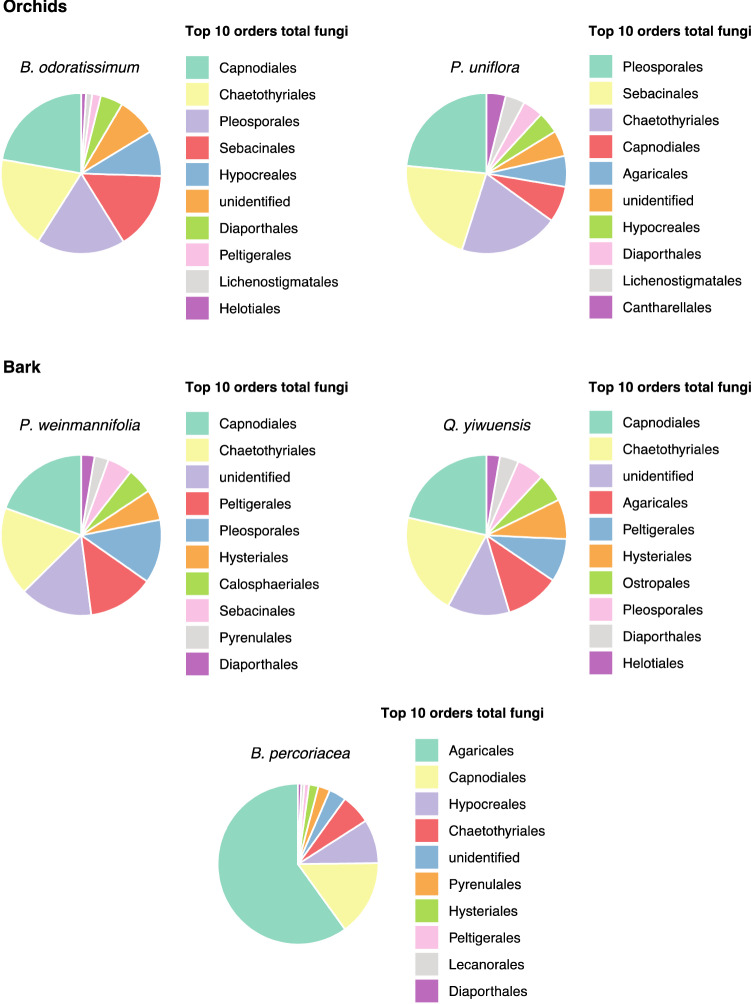
Most abundant orders (top 10) of total fungi present in orchids and bark samples.

The orders Capnodiales, Chaetothyriales, Pleosporales and Sebacinales were the most relative abundant in both orchids representing on average 70% of all reads in *B. odoratissimum* and *P. uniflora*, while the bark samples from *P. weinmannifolia* and *Q. yiwuensis* were predominantly colonized by Capnodiales, Chaetothyriales and unknown fungi, representing a mean 49% of the reads. The most abundant order in the bark of *B. percoriacea* was Sebacinales representing a mean of 59% of total reads (Fig. 2).

**Figure 3:**
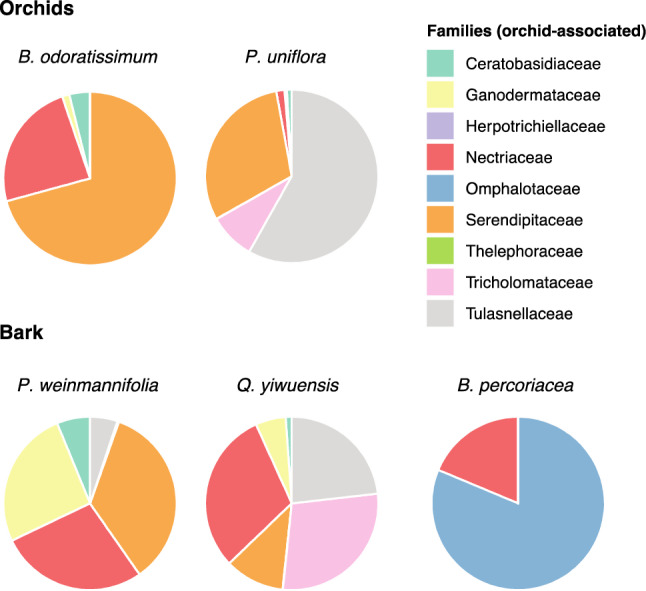
Orchid-associated fungal families detected in orchids and bark samples.

Considering the orchid-associated fungi, we observed that Serendipitaceae (71% of all reads), Nectriaceae (24%) and Ceratobasidiaceae (4%) were the most relative abundant families associated with the orchid *B. odoratissimum*. Similarly, the bark of its host trees also had 35% of reads belonging to Serendipitaceae, 28% to Nectriaceae, and 26% to Ganodermataceae. The orchid *P. uniflora* had 58% of reads belonging to Tulasnellaceae, 30% to Serendipitaceae and 9% to Tricholomataceae, while its phorophyte bark had 30% of reads belonging to Nectriaceae, 28% to Tricholomataceae, and 23% to Tulasnellaceae. The bark of *B. percoriacea* was constituted by 81% of Omphalotaceae and 19% of Nectriaceae (Fig. 3).

### Fungal community composition

**Figure 4:**
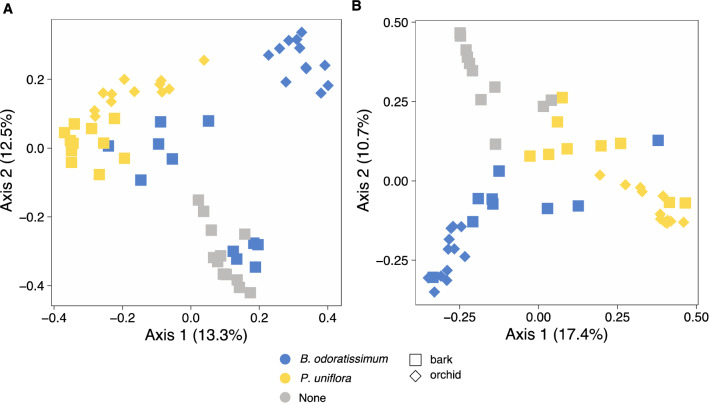
Total (**A**) and orchid-associated (**B**) fungal community structure of *B. odoratissimum* and *P. uniflora* and of the bark from the host tree where the orchids were collected.

Fungal communities were significantly structured by orchid species and host tree on both total (orchid: *R*^*2*^ = 0.19, *F* = 7.04, *P* = 0.001, host tree: *R*^*2*^ = 0.09, *F* = 6.33, *P* = 0.001) and orchid-associated (orchid: *R*^*2*^ = 0.21, *F* = 7.23, *P* = 0.001, host tree: *R*^*2*^ = 0.04, *F* = 3.00, *P* = 0.001) fungal datasets (Fig. 4). Pairwise comparisons indicated that both total and orchid-associated fungal communities were distinct between the two orchids, on the bark of the three tree species, and between any orchid and any tree species (P < 0.050 for all multiple comparisons). Variation partitioning indicated that orchid species explained 16.0% and host tree explained 8.6% of the variance of the total fungal communities, while orchid species explained 18.3% and host tree explained 4.8% of the variance of the orchid-associated fungal communities.

Fungal communities were not statistically different according to the height in the tree where samples were collected in both fungal datasets. Moreover, no significant differences were found between the geographic location of the samples and their fungal communities except for orchid-associated fungi found in orchids in the highest height on the tree (Supplementary Table S2).

### Bark fungal communities

**Figure 5:**
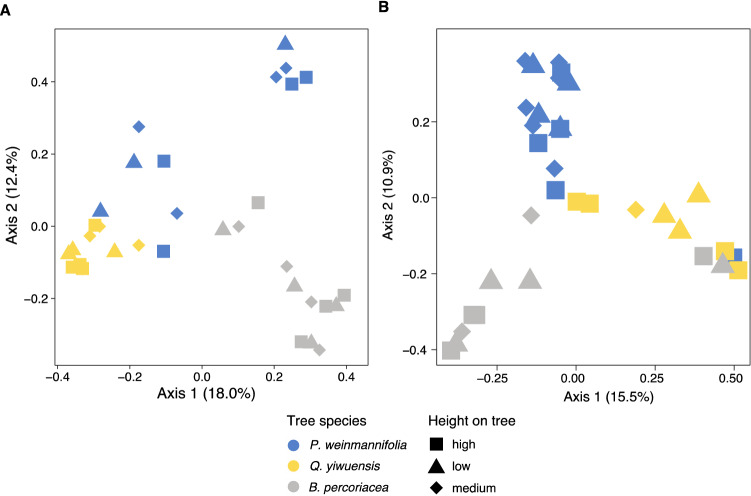
Total (**A**) and orchid-associated (**B**) fungal community structure of the bark from the host tree where the orchids were collected.

The fungal communities in the bark of the three trees were structured by tree species (total fungi: *R*^*2*^ = 0.22, *F* = 4.458, *P* = 0.001; orchid associated: *R*^*2*^ = 0.20, *F* = 3.415, *P* = 0.001), and individual tree (total fungi: *R*^*2*^ = 0.59, *F* = 2.842, *P* = 0.001; orchid-associated: *R*^*2*^ = 0.55, *F* = 2.006, *P* = 0.001), but the height in the tree had no effect (Fig. 5).

### Tree and bark features

The selected trees were in the same height classes: 9.5–11.5 m in *Q. yiwuensis*, 5–15 m in *P. weinmannifolia*, and 5–11.5 m in *B. percoriacea*. Breast height diameters differed significantly: 0.47 m ± 0.22 in *Q. yiwuensis*, 0.35 m ± 0.23 in *P. weinmannifolia* and only 0.07 ± 0.01 in *B. percoriacea*. The latter were thus consistently more slender trees, with only one of the selected *P. weinmannifolia* trees being in the same range (*B. percoriacea* < *Q. yiwuensis*: P < 0.05). The preliminary HPLC analysis revealed the presence of different compounds in the three bark samples representing the three phorophyte species. In particular, the chromatogram (Supplementary Fig. S1) showed two peaks at the retention time of 16 min for the *Q. yiwuensis* (P-tree) sample, which are missing in the *P. weinmannifolia* (B-tree) and *B. percoriacea* (N-tree) samples, whereas one major peak at 27 min was exclusive for the B-tree bark.

Although our samples represented bark from below breast height to 2.5 m, the bark features showed no height trends. Thus, the data from the three heights were averaged to represent the individual tree and mean and standard deviation calculated across 4 individuals of each tree species (Table 1). Bark thickness differed by about a factor of 3 from the thinnest (*B. percoriacea*) to the thickest bark (*Q. yiwuensis* (t-test, P < 0.02)). Bark density was within the same range for all tree species. Water holding capacity was significantly lower in *Q. yiwuensis* than in *P. weinmannifolia* and *B. percoriacea* (t-test, P < 0.004). Bark pH was similar in *P. weinmannifolia* and *B. percoriacea*, but tended to be higher in *Q. yiwuensis* (P < 0.07 and 0.06, respectively).

**Table 1:** Bark features in the 3 analysed species in the study area.

Tree species	Bark thickness	Bark density	Water holding capacity	Bark pH	Maximum profile height (Rt)	Mean profile height Rz (DIN)
	mm	mg/mm^3^	%		mm	mm
*Q. yiwuensis*	11.4 ± 1.7^a^	0.10 ± 0.03	45.82 ± 0.77^b^	6.5 ± 022	10.08 ± 3.37	4.62 ± 1.59 ^a^
*P. weinmannifolia*	7.2 ± 1.6^b^	0.08 ± 0.01	52.74 ± 2.48^a^	6.0 ± 0.36	8.42 ± 4.28	3.62 ± 0.98 ^a^
*B. percoriacea*	4.1 ± 0.8^c^	0.09 ± 0.02	52.40 ± 2.73^a^	6.0 ± 0.34	5.00 ± 1.41	1.52 ± 0.49 ^b^

Number of bark cores 3–6 from each tree, taken at different heights on the tree. Data shown are means and SD of four individual trees (bark thickness, density, WHC). Bark roughness was measured in situ with a 150 mm profile comb. Different lettering indicates significant differences at P ≤ 0.05.

The barks of the two species of trees carrying the target orchid species (*Q. yiwuensis* and *P. weinmannifolia*) were rather coarse with 8–10 mm deep grooves and ridges and were visually quite similar. Both phorophyte species carried many other epiphytes, including several orchid species. The bark of *B. percoriacea*, which did not carry any of the two target orchid species, was significantly smoother (P < 0.01) and had few epiphytes (ferns, mosses and lichens), the only orchids seen being unidentified pseudobulbs of *Eria* sp.

## Discussion

This study significantly contributed to our knowledge of different components of the complex habitat represented by trees that carry epiphytic orchids, and improved our understanding of some aspects of this phorophyte-epiphyte relationship, which up to now, were nearly completely unknown^8,9^. For the first time, we provided a comprehensive characterization of fungal communities colonizing the bark of phorophyte trees, by combing molecular and morphological analyses of culturable fungi, and metabarcoding analyses based on Illumina sequencing. We unveiled a rich culturable fungal community, mostly constituted by ascomycetous strains, which was significantly different between the three investigated tree species, being just 9 out of 74 fungal genera in common for all tree species, and only two genera shared by P- and B-trees, which carried the studied orchids. This clear difference in bark-associated fungi may represent a crucial factor determining the selective preference of *B. odoratissimum* and *P. uniflora* for their phorophytes. Although most of isolated fungi are not considered typical orchid symbionts, at least for adult orchids, some of them have been repeatedly observed to colonise orchid roots in previous studies, raising the hypothesis of a possible trophic role that these fungi can play towards their associated orchid species^27,28^. For instance, ascomycetes belonging to the genus *Fusarium*, which were present on P- and B-trees, while not found on N-trees, have been previously identified as endophytes from tissues of a variety of orchid species, such as *Pecteilis susannae*^29^, *Orchis tridentata*^30^, and *Ophrys bertolonii*^28^. Besides several studies showed that *Fusarium* fungi can stimulate orchid seed germination and seedling growth^31–33^. Therefore, we cannot exclude that the presence of these fungi on the bark of phorophytes could be important for the early stages of orchid development, thus affecting orchid presence on certain trees. Further studies, using seed germination experiments are needed to clarify the identity of fungi that sustain the initial development of the studied orchids. Similar hypothesis could be set up for other ascomycetous fungal species isolated from bark samples in this study, including *Exophiala salmonis*, which has been reported to colonize the roots of various orchid species in several studies^34–36^, as well as *Alternaria* sp., *Nectria* sp. and *Phoma* sp. previously found in the roots of several green terrestrial orchids in Italy^28,30,35,37^. Also among the basidiomycetes isolated from the studied phorophyte trees there are species that deserve further analysis to test their potential role as orchid seed germination supporting fungi, including *Coprinellus* species, which have been previously shown to establish symbiotic relationships with the fully mycoheterotrophic orchid *Epipogium roseum*^38^, and *Marasmius* fungi, which have been identified as the main mycorrhizal partners of *Gastrodia sesamoides*^39^. From the list of bark isolated fungi that deserve to be mentioned as potential species for sustaining juvenile orchid development, the zygomycete *Mortierella alpina* is particularly interesting, because it has been recently found to colonize the roots of an achlorophyllous orchid species, *Chamaegastrodia inverta* in southern China^40^. In the latter study, *Mortierella* strains accounted for 46.8% of total cultured mycelia, being the most commonly isolated fungi from the analysed orchid roots^40^. All the above-mentioned fungi isolated from the bark of the investigated phorophyte species deserve further studies based on both in situ and ex situ seed germination experiments to test whether or not they play a functional role in the early development of the two analysed orchid species. Our attempts of bark culturable fungi isolation did not yield any strain of rhizoctonioid fungi in Ceratobasidiaceae, Sebacinaceae, and Tulasnellaceae, which represent the majority of both terrestrial and epiphytic green orchids mycobionts^41–44^. To our knowledge, the sole previous study on fungi isolated from the bark of orchid phorophyte trees^23^ reported *Rhizoctonia* as one of the most abundant fungal *taxa*, together with *Penicillium* and *Trichoderma*. While the results of Tremblay and co-authors^23^ on the latter two genera are consistent with our findings, their successful isolation of *Rhizoctonia* strains, which is in contrast with our study, may be explained by the use of a specific *Rhizoctonia* isolation medium^23^, even though *Rhizoctonia*-like fungi are known to normally grow on PDA^45^. However, in our study, the presence of unculturable fungi was detected using a metabarcoding approach, which revealed the total diversity of fungal communities growing on the phorophyte bark and on the orchid roots, thus confirming the importance of high-throughput next generation sequencing analyses for large-scale ecological studies of fungi^46,47^.

Illumina sequencing showed that the identity of the orchid-phorophyte bark pair *B. odoratissimum*-*P. weinmannifolia* and *P. uniflora-Q. yiwuensis* explained 16.0% and 18.3% of the variance of the fungal community structure for both total and orchid-associated fungi. Despite fungal communities also being structured by host tree, identity of the pair orchid-bark explained higher variance than the distinction between orchid and bark samples. In addition, fungal communities associated with the bark of *B. percoriacea* were very different to those of the other two tree species which harboured orchids. This suggests some affinity between the fungal communities of the bark of particular host trees and particular orchids and could help explaining why each orchid was only found to grow on a particular phorophyte. Based on the total fungi, fungal communities of *P. uniflora* orchids and their host tree species *Q. yiwuensis* showed considerable similarity. Such similarity was not present between *B. odoratissimum* and its tree *P. weinmannifolia* (Fig. 4A). However, in the orchid-associated fungal communities, substantial similarity between orchids and host tree was detected (Fig. 4B), suggesting that the orchid-host tree association was influenced by the fungal communities of the host tree bark. This was also reflected in the taxonomic composition of the orchid-host tree communities: besides Serendipitaceae, Tulasnellaceae and Tricholomataceae were well-represented in the fungal diversity of both *P. uniflora* and the bark of its phorophyte. These two families were respectively poorly represented and absent on the bark of *P. weinmannifolia,* and absent from the roots of *B. odoratissimum* orchids growing on the latter tree. Conversely, *B. odoratissimum* orchids and *P. weinmannifolia* showed overlap in Serendipitaceae, Gymnodermataceae, and Nectriaceae. Fungi of the latter family were also present on the non-host tree *B. percoriacea,* and thus unlikely contributed to the *B. odoratissimum–P. weinmannifolia* specificity. These patterns suggested that presence of particular orchid associated fungal families on the bark of the phorophytes may contribute to the orchid-tree specificity observed in these two pairs of orchid-host trees. Fungal communities of both orchids and bark were not different depending on the height on the tree. Fungal communities were specific of each bark, and the height on the tree did not impose any restriction to the assembly of particular communities for both total fungi and orchid-associated fungi. Thus, the height on the trees where these orchids germinate may not be dependent on availability of fungal communities.

Considering that both analysed orchids, *P. uniflora* and *B. odoratissimum* grew within the quite limited investigated area and the rain of their seeds must fall indiscriminately on all trees on the studied hilltop, two questions present themselves: Why was *B. percoriacea* not used as phorophyte, and why did each of the two orchid species avoid the phorophyte of the other? Furthermore, the differing fungal communities on the three tree species require an explanation. Just as the seed rain, the rain of fungal spores and propagules would not be able to discriminate the tree trunks. Nevertheless, few fungal *taxa* established on all three tree species, and a considerable part of them was exclusively found on one species of tree. This observation is an important result of this study, as it indicates an interaction between factors in the bark of trees and fungal community composition. This reflects a profuse biodiversity that has escaped attention previously. Concerning the unsuitability of *B. percoriacea* as phorophyte, the individual trees available in this species were very slender, compared to the other two tree species. This means that the colonizable area for epiphytes was much less than in the other tree species under study. Previous studies have reported on the importance of tall and large trees to provide substrate for orchid species^2,16^, while a more general work on epiphyte-phorophyte network have shown that the size of trees influenced the network structure, and the distribution of epiphyte species differed between the phorophyte ecological zones^4^. However, *B. percoriacea* trees had large areas of unoccupied bark, which suggested that mutual competition between epiphytes was not an obstacle to the establishment of orchid seeds. *Beilschmiedia percoriacea* had smooth bark which could be due to less thickness growth in these trees, and a bark structure that yields to tangential stretching instead of cracking and producing secondary cork cambia. This low bark profile might constitute less variability in microsites, thus offering fewer microhabitats for epiphytes^3,14^. In contrast, the fungal community was more diverse in *B. percoriacea* than elsewhere, as confirmed by both isolation methods and metabarcoding analysis. However, looking at the identity of fungi colonizing *B. percoriacea*, which was significantly different from *Q. yiwuensis* and *P. weinmannifolia* fungal diversity, this speaks in favour of a lack of suitable orchid mycobionts as an explanation of the poor performance of the Neutral-tree as orchid phorophyte. Concerning the fact that *Q. yiwuensis* was preferred by one orchid species (*P. uniflora*) and *P. weinmannifolia* by another (*B. odoratissimum*), this tells us that the two orchid species probably have contrasting requirements for their germination niche^9^. The two phorophyte species were in the same size range, and there were few distinguishing bark features. However, the bark was thicker in *Q. yiwuensis* which indicated a stronger cork development on them than on *P. weinmannifolia*. Consistent with this observation, the water holding capacity was lower. Besides that, bark pH was higher in *Q. yiwuensis* than in *P. weinmannifolia*. The two latter traits might affect seed germination directly. We might hypothesize that water availability is more critical to *B. odoratissimum* than it is to *P. uniflora*, and thus *B. odoratissimum* cannot establish on the drier bark of *Q. yiwuensis*. A previous work performed in two tropical forests in southeast Mexico has shown a positive relationship between water-storage-capacity, bark porosity, and orchid phorophyte preference^12^. Unfortunately, we could not test this hypothesis experimentally, as we did not have access to seeds. Likewise, we could hypothesize that *P. uniflora* is unable to germinate on a substrate with low pH, the critical threshold being somewhere above pH = 6.0. Again, an experimental sowing would be required to test this idea. Moreover, given that water availability and pH are two of the most influential environmental factors for fungal development^48^, it is very likely that these parameters played a major role in driving the differences between fungal communities colonizing the studied phorophyte species, and in particular the presence and abundance of orchid suitable mycobionts. Differences in chemical compounds present in the bark may also affect the fungal communities growing on each tree species, as suggested by the preliminary analysis performed in this study. More detailed analyses are necessary to clarify the chemical composition of the bark from the three phorophytes. The bark observations and measures did not indicate any connection between bark roughness and the mutually exclusive occurrences of *P. uniflora* and *B. odoratissimum* on their respective phorophytes, but it seems reasonable to assume that the scarcity of epiphytes on *B. percoriacea* is connected with its smoother bark. Our finding is in agreement with the recent study, in which Zarate-García and collaborators^12^ described bark decoration patterns and bark fissuring, using a new protocol based on image processing of light micrographs. They also found that bark decoration and fissuring were not explaining orchid preference for phorophytes^12^.

In conclusion, our study provided new insights on the environmental factors affecting the relationship between epiphytic orchids and phorophyte trees. Our analyses of numerous biotic and abiotic elements characterizing the different analysed phorophyte habitats and orchid species, including bark and orchid root fungal diversity, disentangled the effect of the different components involved in the phorophyte-orchid interaction on the preference showed by the studied orchids for their distinct host trees. In particular, our results showed that fungal communities could represent a fundamental factor determining orchid specificity toward particular phorophyte trees. Bark features, such as water holding capacity and pH, could affect the orchid preference for a certain phorophyte both directly, by influencing the orchid seed germination, and indirectly, by shaping the fungal communities available on the bark of different tree species, which are essential for the orchid establishment and growth.

## Methods

### Study site and species

**Figure 6:**
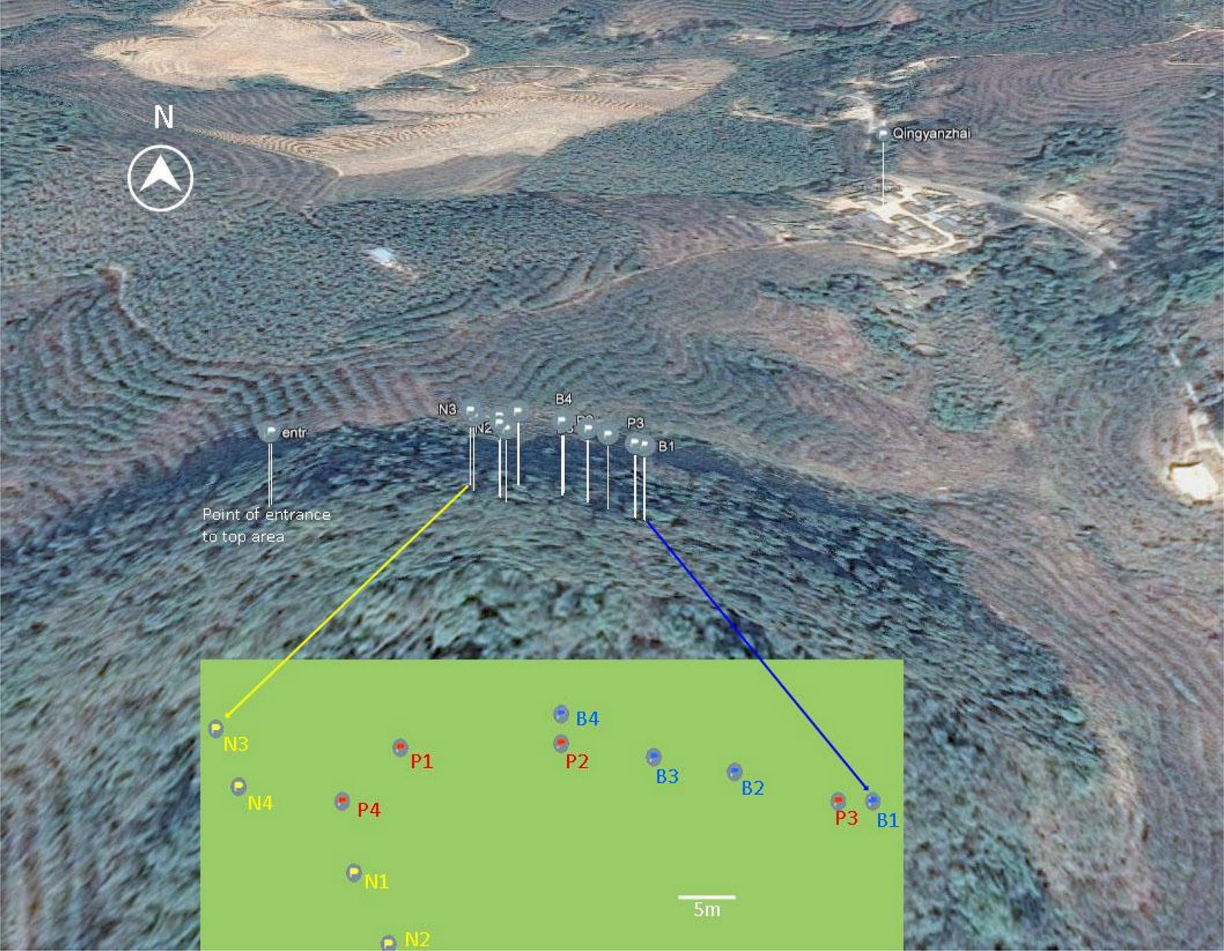
Map of the study site with approximate position of analysed trees (aerial perspective from Google Maps 2018). GPS positions were obtained less than 1 m from the tree trunks. The distance from N3 to B1 is approximately 60 m.

The sub-tropical forest analysed in this study is located in China, Yunnan, Xishuangbanna, Mengla county, Village Quingyanzhai (#94) N 21.802068, E 101.380214, geodetic datum WGS84 (Fig. 6). The site is characterized by a rocky outcrop rising 30–50 m over surrounding rubber plantations, harbouring about 20 ha of relict dry tropical forest. The outcrop sides are steep and mainly covered with bamboo. The top area is colonized by shrubs and 10–15 m high trees (a few trees on the slopes are much higher). The most conspicuous species is *Quercus yiwuensis* Y.C. Hsu & H.W. Jen*.* In March 2017 we selected four individual trees of *Q. yiwuensis*, and an equal number of *Pistacia weinmannifolia* Franch, and *Beilschmiedia percoriacea* C.K. Allen that were also numerous on the site. Plants were identified by the authors in the field and labelled. Botanical specimens were deposited in the School of Pharmaceutical Science and Technology, Tianjin University, Tianjin, China.

*Q. yiwuensis* was designated P-tree (P1, P2, P3, P4) because we consistently found the orchid *Panisea uniflora* Lindl. growing on this phorophyte species. *P. weinmannifolia* was designated B-tree (B1, B2, B3, B4) because it harboured the orchid species *Bulbophyllum odoratissimum* (Sw) Lindl. (Supplementary Fig. S2 a-d). On *P. weinmannifolia* trees no *P. uniflora* was observed, while *B. odoratissimum* was never found on *Q. yiwuensis* trees*.* Both tree species were richly colonized by several other orchid species. *Beilschmiedia percoriacea* trees were designated neutral tree, N-tree (N1, N2, N3, N4), because neither of the two target orchid species grew on them. The latter tree species carried several lichens and a single fern species (*Lepisorus* sp.), but only in one instance was observed to carry an orchid epiphyte (*Coelogyne* sp.).

GPS positions of investigated trees were obtained less than 1 m from the trunk (Fig. 6). Accuracy is about 3 m. Accuracy in altitude readings is about 100 m. Distance between degrees of latitude is 111 km. At N 21.78978 the distance between degrees of longitude is 103 km, which means that the last digit in the 5-digit decimal degrees corresponds to 1.11 m in latitude and 1.03 m in longitude.

The trees were labelled with different colours as follows:

- P-trees (carrying *P. uniflora* and other epiphytes, but not *B. odoratissimum*), identified as *Q. yiwuensis*, with red labels (P1 N 21.79880, E 101.37909, 1073; P2 N 21.79882, E 101.37923, 1072; P3 N 21.79878, E 101.37947, 1074; P4 N 21.79878, E 101.37904, 1073).
- B-trees (carrying *B. odoratissimum* and other epiphytes, but not *P. uniflora*), identified as *P. weinmannifolia*, with blue labels (B1 N 21.79878, E 101.37950, 1074; B2 N 21.79880, E 101.37938, 1078; B3 N 21.79881, E 101.37931, 1083; B4 N 21.79884, E 101.37923, 1076).
- N-trees (carrying epiphytes, but neither *B. odoratissimum* nor *P. uniflora*), identified as *B. percoriacea*, with yellow labels (N1 N 21.79873, E 101.37905, 1064; N2 N 21.79868, E 101.37908, 1072; N3 N 21.79883, E 101.37893, 1071; N4 N 21.79879, E 101.37895, 1071).
- The point of access to the outcrop top area was located at the Western edge (N 21.79880, E 101.37827, 1058, Fig. 6).

### Sampling

For each of the twelve selected trees, breast height circumference (BH = 130 cm above ground) was measured. Approximate total height was determined by Nikon Laser Forestry Pro or estimated if sighting lines were interfered by other vegetation.

The lowermost individual of the target orchid species was recorded in relation to BH. Bark samples were collected, and bark features recorded at BH, by target orchid, and 50 cm above target orchid or BH, whichever was highest point. In N-trees, where there were no target orchids, sampling was thus at BH, BH + 50 cm, and BH + 100 cm.

Sampling on each tree involved approximately 12 cm^2^ bark cut out with a sterile knife and rubber gloves to prevent cross-contamination, for pH-analysis, metabarcoding, fungal isolation and chemical analysis. Besides, 3 bark cores were taken by trephor sampler (16 mm, 2 mm diam., Costruzioni Meccaniche Carabin Carlo) for water holding measurement.

Roots of target orchids were sampled, from three adult individual plants on each P- and B-tree. No permissions were necessary to collect plant samples, using a protocol that avoided plant damages. All plants were left in the exact location where they were found in the sampling site, after collecting the small portions of bark and root material for the study. All experiments including the collection of plant material in this study are in compliance with relevant institutional, national, and international guidelines and legislation.

All fresh material collected from the sampling site was first kept in cool boxes, brought to the laboratory, and processed within three days.

### Fungal isolation from bark

For each sample, half of the bark material and orchid roots were kept at − 80 °C for subsequent metabarcoding analysis. The rest of bark (about 2 g for each sample) was immediately processed for fungal isolation. The large bark portions were ground into powder using a sterile mortar and pestle; 5 ml were reserved for pH measurement, while the rest was suspended in a final volume 50 ml sterile water solution in a sterile centrifuge tube. The tube was shaken with Vortex vibration meter thoroughly and solution aliquots were spread homogenously onto isolation medium plates. For each bark sample, aliquots of 500, 300, 200, and 100 μl, were spread per triplicate to one plate each of PDA (Potato Dextrose Agar) medium, containing ampicillin (50 μg/mL) and streptomycin (50 μg/ml) to inhibit bacterial growth^49,50^. A diluted solution was also made by mixing 1 ml of the original solution with 9 ml sterile water and plated. Petri dishes were incubated at room temperature (23–25 °C) in the dark for up to 2 months to allow the development of slow-growing mycelia. Fast growing fungal strains started to grow after about two days. Colonies showing different morphology and appearance were transferred to fresh plates to obtain pure cultures. In the following days, other slower growing mycelia were available in the Petri dishes and were also regularly picked up and isolated onto new PDA plates every 2 days. All isolated fungal strains were stored at 4 °C for further analysis. All strains were deposited in the LP Culture Collection (personal culture collection held in the laboratory of Prof. Lorenzo Pecoraro), at the School of Pharmaceutical Science and Technology, Tianjin University, Tianjin, China.

### Molecular and morphological analysis of bark culturable fungi

The identification of isolated fungal colonies was performed using DNA sequencing combined with microscopy. Total genomic DNA from isolated fungi was extracted following the cetyltrimethyl ammonium bromide (CTAB) method modified from Doyle and Doyle^51^. Fungal ITS regions were PCR-amplified using the primer pair ITS1F/ITS4^52^ following the procedure described in Pecoraro et al.^37^ for PCR reaction, thermal cycling, and purification of PCR products. Controls with no DNA were included in every amplification experiment in order to test for the presence of laboratory contamination from reagents and reaction buffers. Purified DNA amplicons were sequenced with the same primer pair used for amplification. DNA sequencing was performed at the GENEWIZ Company, Tianjin, China.

Sequences were edited to remove vector sequences and to ensure correct orientation and assembled using Sequencher 4.1 for MacOsX (Genes Codes, Ann Arbor, MI). Sequence analysis was conducted with BLAST searches against the National Center for Biotechnology Information (NCBI) sequence database (GenBank; http://www.ncbi.nlm.nih. gov/BLAST/index.html) to determine the closest sequence matches that enabled taxonomic identification. DNA sequences were deposited in GenBank (Accession Nos. MW603206 – MW603451). Fungal morphological characters (hyphae, pseudohyphae, conidiophores, conidia, poroconidia, arthroconidia, etc.) were examined using a Nikon ECLIPSE Ci microscope for the identification of isolates following the standard taxonomic keys^53–57^.

### Assessment of bark and orchid associated fungal community using Illumina sequencing

Bark and orchid root samples were pulverized in a sterile mortar, and genomic DNA was extracted using the FastDNA® Spin Kit as described by the manufacturer (MP Biomedicals, Solon, OH, USA)^58,59^. In total, this resulted in 60 DNA samples, including 36 from bark (3 sampling points for each tree × 12 trees) and 24 from orchid roots (3 orchid individuals sampled on each P- and B-tree × 8 trees; the 4 individual N-trees were not used for orchid sampling because they did not carry the study orchid species). Subsequently, amplicon libraries were created using two primer combinations targeting the internal transcribed spacer 2 (ITS-2): ITS7F and ITS4R^60^ was used as universal fungal primer pair to target nearly all fungal species, while ITS3^61^ and ITS4OF^62^ was used to more specifically target orchid mycorrhizal fungi. Previous research has shown that most universal fungal primers have multiple mismatches to many species of the orchid-associating basidiomycetes, in particular in Tulasnellaceae family^46,58,63^. Since the goal of the present work was to analyse the total fungal community in the orchid-phorophyte environment (bark and orchid roots), as well as more specifically detect the orchid mycorrhizal fungi in the studied samples, it was necessary to combine two different primer pairs to characterise the whole investigated fungal diversity^47,64–66^. Polymerase chain reaction (PCR) amplification was performed in 50 μl reaction volume, containing 38 μl steril distilled water, 5 μl 10 × buffer (100 mM Tris–HCl pH 8.3, 500 mM KCl, 11 mM MgCl_2_, 0.1% gelatin), 1 μl of dNTP mixture of 10 mM concentration, 0.25 μM of each primer, 1.5 U of RED TaqTM DNA polymerase (Sigma) and approximately 10 μg of extracted genomic DNA. PCR conditions were as follows: 1 cycle of 95 °C for 5 min initial denaturation before thermocycling, 30 cycles of 94 °C for 40 s denaturation, 45 s annealing at various temperatures following Taylor and McCormick^62^, 72 °C for 40 s elongation, followed by 1 cycle of 72 °C for 7 min extension. To minimize PCR bias, three PCRs were pooled for each sample. The resulting PCR products were electrophoresed in 1% agarose gel with ethidium bromide and purified with the QIAEX II Gel Extraction Kit (QIAGEN). Amplicon libraries were generated using the NEB Next Ultra DNA Library Prep Kit for Illumina (New England Biolabs, USA) following the manufacturer's instructions to add index codes. Samples were sequenced using the Illumina MiSeq PE 250 sequencing platform (Illumina Inc., San Diego, CA) at Shanghai Majorbio Bio‐Pharm Technology Co., Ltd. (Shanghai, China).

### Bioinformatics of fungal sequences

Sequences originated from the total (ITS7F and ITS4R primers) and orchid-associated (ITS3 and ITS4OF primers) fungi datasets were processed separately. Raw reads were merged with a minimum overlap of 30 nucleotides, and the primer sequences were trimmed off. Subsequently, reads were filtered by discarding all sequences with expected error > 1. The quality-filtered reads were denoised using the UNOISE3 algorithm^67^ to create zero-radius operational taxonomic units (zOTUs), with chimera removal. All the steps were performed using USEARCH v.11^68^. Raw sequences have been deposited in the Sequences Read Archive (SRA) of NCBI as BioProject ID PRJNA702612. The fungal zOTUs were assigned to taxonomic groups using the Blast algorithm by querying against the UNITE + INSD fungal ITS database (version 7.2, released on 10 October 2017)^69^ using the *sintax* algorithm with 0.8 cutoff^70^. The zOTUs originated with the orchid-associated fungal primers were manually screened for possible orchid-associated mycorrhizal families based on the information provided in Table 12.1 in Dearnaley et al.^71^, and only these were retained for further analysis in this dataset.

To attempt removing spurious counts due to cross-talk (assignment of reads to a wrong sample) we removed all the zOTUs represented by less than 0.02% of reads in each sample, which is more conservative than previous error estimates^72^. The datasets were rarefied to the minimum sequencing depth (23,419 for total fungi and 13,074 for orchid-associated fungi), zOTUs present in less than three samples and low abundant zOTUs (with relative abundance < 0.1%) were removed from further analyses. The filtering steps resulted in removing two and six samples from the total and orchid-associated fungi datasets, respectively.

### Fungal communities

We tested for differences in richness between orchid and bark samples (host tree), and for the interaction between host tree and orchid species using ANOVA. We performed pairwise comparisons using *t-test* with Bonferroni corrections to assess differences in richness between the two orchid species (*B. odoratissumum* and *P. uniflora*) and the barks from the three tree species (*P. weinmannifolia, Q. yiwuensis* and *B. percoriacea*).

We tested for the effect of orchid species and host tree on the total and orchid-associated fungal community structure with two-way PERMANOVA analyses with 999 permutations using the function *adonis2* in *vegan* R package^73^ on the Bray–Curtis dissimilarity matrix calculated on the Hellinger transformed count data. Pairwise comparisons were performed using the *RVAideMemoire* R package with Benjamini–Hochberg corrections^74^. Variance explained by orchid species and host tree was investigated with variation partitioning using the *vegan* R package. We also investigated whether fungal communities varied according to the height on the tree where they were collected. In addition, to understand whether each tree had specific fungal communities on the bark, we tested for the effect of tree species, individual tree and height in tree on the total and orchid-associated fungal communities only between bark samples using one-way PERMANOVAs.

To test whether the geographic location of the sampled trees had an influence on the fungal community composition of the orchids and bark, we computed Mantel test correlation tests between the distance among sampled trees, calculated with the *geosphere* R package^75^ on the geographic coordinates, and the Bray–Curtis dissimilarity matrix of fungal communities. Because we collected samples at three heights in each tree, we computed a separated Mantel test for each height.

### Bark chemical analysis

In the laboratory, small chips were taken from selected bark samples for pilot chemical analysis, comprising one pooled sample from each tree species (P-, B-, N-tree). They were carefully cleaned from every lichen and moss residual, then dried overnight at 50 °C and ground in a mortar. Bark content was subsequently extracted in methanol (50 ml); the ground material was soaked, vortexed and sonicated for 30 min in an ultrasonic bath. The mixture was centrifuged at 10,000 rpm for 15 min. The supernatants were collected and filtered. The extraction was repeated three times on the precipitate. The three supernatants for each bark sample were finally merged in a single tube and vacuum-dried to afford the crude extracts. The extract was dissolved in 2 mL methanol, and centrifuged. Analytical HPLC was conducted with a Waters system (Waters e2695-2998, Photodiode Array Detector, Milford, MA, USA) using a C18 column (Hypersil GOLD C18 column; 5 μm; 4.6 × 250 mm, with a flow rate of 1.0 ml/min; from 20% MeOH in H2O with 0.1% HCOOH to 100% MeOH for 30 min, followed 100% MeOH for 10 min; 1.0 ml/min; The UV detection wavelengths were set at 230 and 254 nm, respectively).

### Bark pH

The ground bark (particle size < 2 mm) was weighed into portions of 150, 100, 50 mg, or less, as availability allowed. They were subsequently immersed in milliq water, amounting to 0.04 ml per mg bark, shaken 60 rounds/min for 60 min, kept stationary to settle for 60 min and measured with an Unisense pH microelectrode system.

### Water holding capacity of bark

One or two cores from each tree and height with intact inner and outer bark were, after removal of any xylem parts, selected for water holding capacity measurement. Immediate weight, wet weight after soaking in dd water for 24 h, and dry weight determined after desiccation at 50 °C in oven overnight. While in soaked condition, the core lengths were measured in dissection microscope at 12 × magnification to obtain a measure of bark thickness and core volume. Water holding capacity was calculated as (WW-DW)*100/WW.

### Assessment of bark roughness

Bark roughness of each analysed tree was recorded at the three bark sampling points described above, by determining a vertical bark surface profile, using a Yamano 150 mm contour gauge (Yamano Seisakusho, Japan) held parallel to the length axis of the tree trunk, the shape of which was subsequently photographed. Simple surface roughness parameters usable at all scales were measured for numerical assessment: Contour length, ratio contour length/evaluation length, maximum height of profile (Rt) and maximum heights (Rti) within 5 sampling intervals; average maximum height of the profile Rz(Din) was calculated (Supplementary Figs. S3 and S4) (DIN 4768 1990 Deutsches Institut fur Normung E.V.)^76^. Contour length was measured with the image processing program ImageJ 1.50i (2016).

## Supplementary Information

Supplementary Information.
