# Supplementary material for: Fungal diversity driven by bark features affects phorophyte preference in epiphytic orchids from southern China
Source: Sci Rep. 2021 May 28;11:11287. doi: 10.1038/s41598-021-90877-1 (PMC8163780; doi:10.1038/s41598-021-90877-1)
Supplement: Supplementary file 1 — Supplementary Information. [file 41598_2021_90877_MOESM1_ESM.docx]

**Fungal diversity driven by bark features affects phorophyte preference in epiphytic orchids from southern China.**

Lorenzo Pecoraro^1, *^, Hanne N. Rasmussen^2^, Sofia I.F. Gomes^3^, Xiao Wang^1^, Vincent S.F.T. Merckx^3^, Lei Cai^4^, and Finn N. Rasmussen^5^

*^1^School of Pharmaceutical Science and Technology, Tianjin University, Tianjin 300072, China*

*^2^Institute for Geoscience and Nature Management, University of Copenhagen, 1958 Frederiksberg C, Denmark*

*^3^Naturalis Biodiversity Center, 2332 AA Leiden, The Netherlands*

*^4^State Key Laboratory of Mycology, Institute of Microbiology, Chinese Academy of Sciences, Beijing 100101, China*

*^5^Natural History Museum of Denmark, University of Copenhagen, 1350 Copenhagen K, Denmark*

*lorenzo.pecoraro@tju.edu.cn

**Supplementary Figure S1**. HPLC chromatogram of bark sample compounds extracted with methanol from the three analyzed phorophyte species (P-, B-, N-trees). Compounds were visualized under UV-light (λ = 230 nm and 254 nm).

**Supplementary Figure S2.** Sampling. *Pistacia weinmannifolia* (B-tree) (a) and its associated epiphytic orchid *Bulbophyllum odoratissimum* (b); *Quercus yiwuensis* (P-tree) (c) and its orchid *Panisea uniflora* (d).

a b


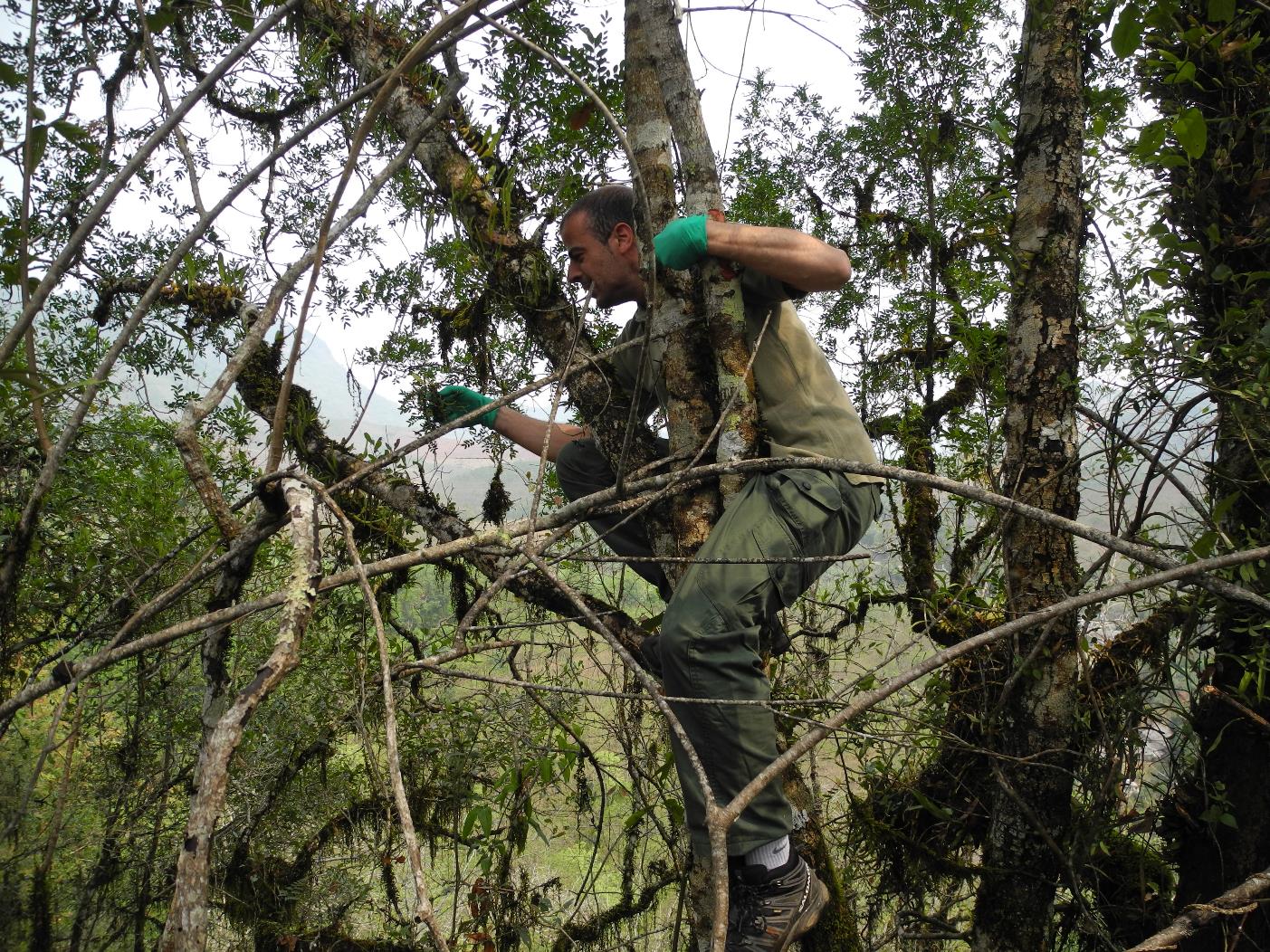

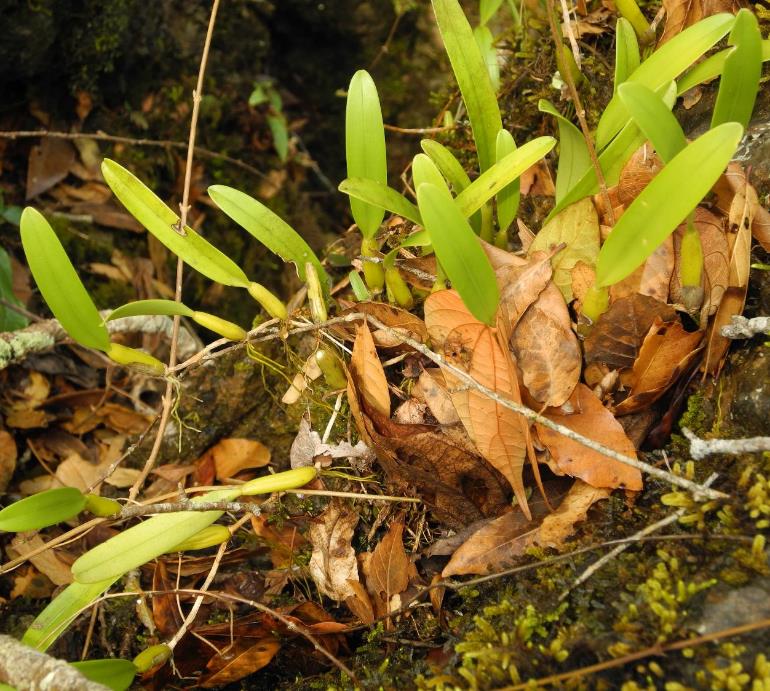


c d


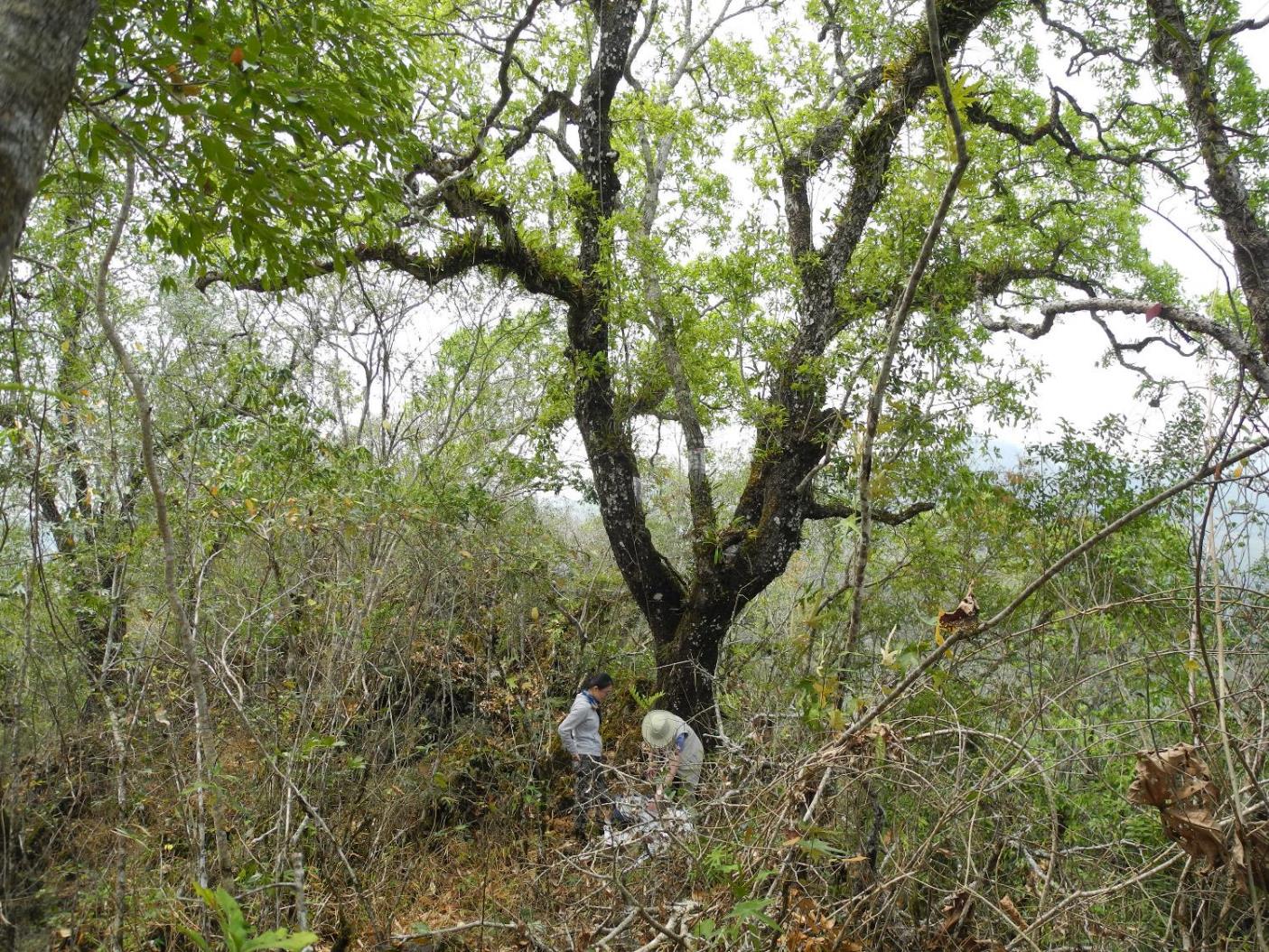

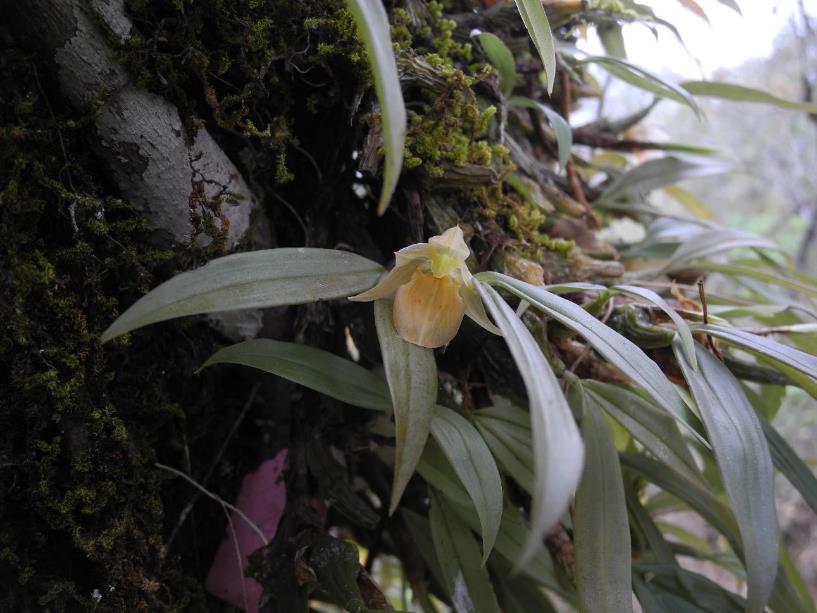


**Supplementary Figure S3.** Measuring bark roughness.


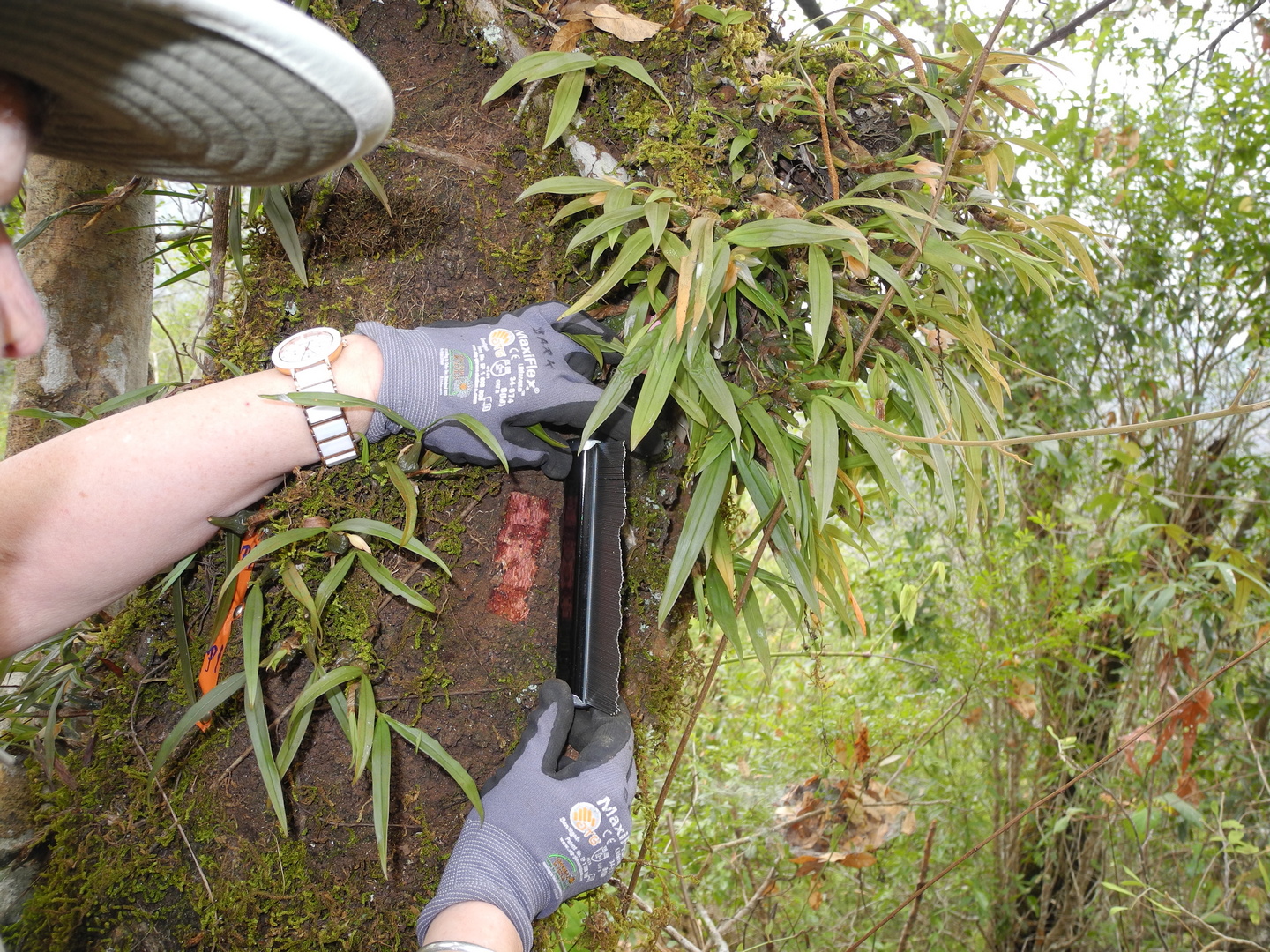


**Supplementary Figure S4.** The bark profile recorded and measured on *Q.* *yiwuensis* P3, 50 cm above the lowermost individual of the target species (*P. uniflora*). The contour gauge is photographed with 1 mm graph paper as background.

Parameters observed for assessing bark roughness: The evaluation length is subdivided into 5 sampling lengths, the vertical distance between the highest and lowest points of the profile with each sampling length (Rt1-5) and the vertical distance between the highest and lowest point of the entire profile (Rt) are measured and the average of the successive values of Rti calculated.


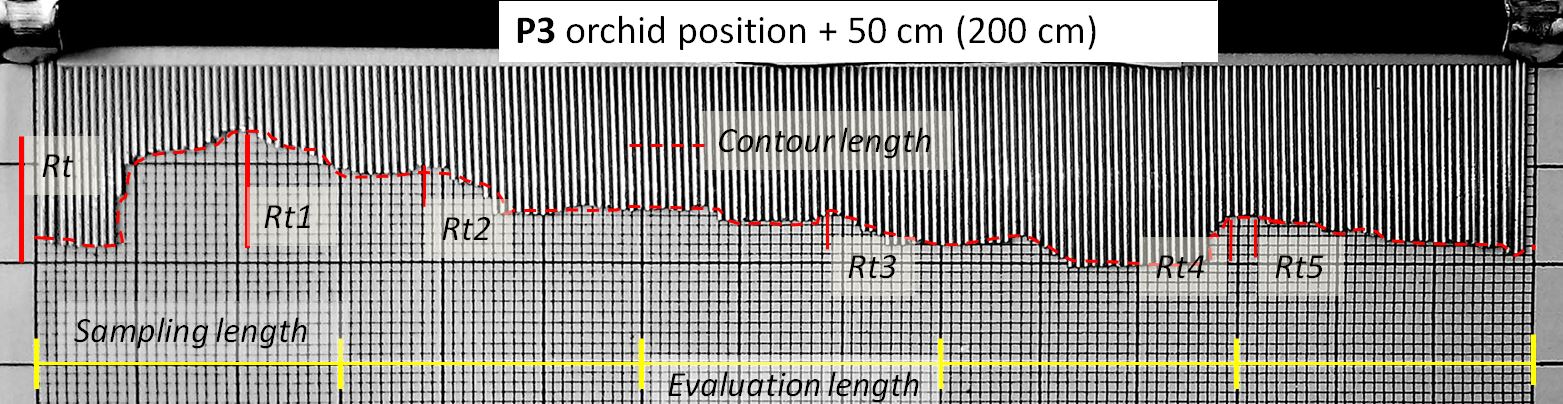


**Supplementary Table S1**. Fungal diversity molecularly detected in the bark of the three analyzed phorophyte species, from DNA extracted from isolated fungi.

BLAST search closest matches of fungal internal transcribed spacer DNA sequences amplified from bark samples. In fungal strain code, first letters indicate the tree species (N=N-tree=*B. percoriacea*, P=P-tree=*Q.* *yiwuensis*, B=B-tree=*P. weinmannifolia*) where the fungus was found, numbers (from 1 to 4) the tree individual, roman numbers (I, II, III) the increasing sampling height, numbers and letters in brackets the isolation plate-dilution and the isolated fungal colony. Sample GenBank accession codes, accession codes for the closest GenBank matches, sequence identity, and overlap of each match are reported.

| **Fungal strain code** | **Number** | **GenBank code** | **Best BLAST match(es)** | **Accession code** | **Overlap length** | **% match** |
| --- | --- | --- | --- | --- | --- | --- |
| N1 I (2-1 a) | 1 | MW603206 | Fungal endophyte | [KR016657](https://www.ncbi.nlm.nih.gov/nucleotide/969821874?report=genbank&log$=nucltop&blast_rank=1&RID=V1MDJ6UP014) | 957 | 99% |
|  |  |  | *Xylaria* sp. | [HQ435669](https://www.ncbi.nlm.nih.gov/nucleotide/315940063?report=genbank&log$=nucltop&blast_rank=25&RID=V1MDJ6UP014) | 941 | 98% |
| N1 I (2-1 b) | 2 | MW603207 | *Chaetothyrium* sp. | [MF136584](https://www.ncbi.nlm.nih.gov/nucleotide/MF136584.1?report=genbank&log$=nucltop&blast_rank=1&RID=5V2W1C2D014) | 640 | 89% |
| N1 I (2-2) | 3 | MW603208 | *Cladosporium ramotenellum* | [LN834386](https://www.ncbi.nlm.nih.gov/nucleotide/893146032?report=genbank&log$=nucltop&blast_rank=1&RID=V4VCBYRH014) | 931 | 99% |
| N1 II (2-1 a) | 6 | MW603209 | *Beauveria bassiana* | [KT280276](https://www.ncbi.nlm.nih.gov/nucleotide/959587089?report=genbank&log$=nucltop&blast_rank=1&RID=V1MS3VH8014) | 963 | 99% |
| N1 II (2-1 b) | 7 | MW603210 | Fungal sp. | [FJ025163](https://www.ncbi.nlm.nih.gov/nucleotide/205277714?report=genbank&log$=nucltop&blast_rank=1&RID=V4VHSMTX014) | 970 | 99% |
|  |  |  | *Nectria* sp. | [FJ025156](https://www.ncbi.nlm.nih.gov/nucleotide/205277707?report=genbank&log$=nucltop&blast_rank=2&RID=V4VHSMTX014) | 963 | 99% |
| N1 II (2-1 c) | 8 | MW603211 | *Biscogniauxia* sp. | [KP306931](https://www.ncbi.nlm.nih.gov/nucleotide/KP306931.1?report=genbank&log$=nucltop&blast_rank=1&RID=5V8USCGW014) | 1170 | 99% |
| N1 II (2-2 a) | 9 | MW603212 | *Hypocrea lixii* | [JX173851](https://www.ncbi.nlm.nih.gov/nucleotide/401878940?report=genbank&log$=nucltop&blast_rank=1&RID=V1MY03RW014) | 1137 | 99% |
| N1 II (2-2 b) | 10 | MW603213 | *Nectria* sp. | [FJ025156](https://www.ncbi.nlm.nih.gov/nucleotide/205277707?report=genbank&log$=nucltop&blast_rank=1&RID=V4VTA2ND016) | 968 | 99% |
| N1 II (2-2 c) | 11 | MW603214 | *Xylaria* sp. | [JQ341083](https://www.ncbi.nlm.nih.gov/nucleotide/407378252?report=genbank&log$=nucltop&blast_rank=1&RID=V4VYF1K7014) | 1018 | 99% |
| N1 II (2-2 d) | 12 | MW603215 | *Virgaria nigra* | [AB670716](https://www.ncbi.nlm.nih.gov/nucleotide/485820442?report=genbank&log$=nucltop&blast_rank=1&RID=V4W2YN67014) | 1090 | 99% |
| N1 II (2-3) | 13 | MW603216 | *Pestalotiopsis microspora* | [KX755256](https://www.ncbi.nlm.nih.gov/nucleotide/1068083545?report=genbank&log$=nucltop&blast_rank=1&RID=V1N1DTJY014) | 1020 | 99% |
| N1 III (1-1-1) | 14 | MW603217 | Uncultured fungus | [GQ999256](https://www.ncbi.nlm.nih.gov/nucleotide/299767664?report=genbank&log$=nucltop&blast_rank=1&RID=VC094MT1014) | 1245 | 99% |
|  |  |  | *Letendraea helminthicola* | [KU529827](https://www.ncbi.nlm.nih.gov/nucleotide/1044974964?report=genbank&log$=nucltop&blast_rank=4&RID=VC094MT1014) | 1170 | 99% |
| N1 III (1-1-2) | 15 | MW603218 | *Pestalotiopsis oxyanthi* | [KP900246](https://www.ncbi.nlm.nih.gov/nucleotide/829098570?report=genbank&log$=nucltop&blast_rank=1&RID=V1NBF2UM014) | 924 | 99% |
| N1 III (1-1-3 a) | 16 | MW603219 | *Psiloglonium* sp. | [KY378959](https://www.ncbi.nlm.nih.gov/nucleotide/1123883246?report=genbank&log$=nucltop&blast_rank=1&RID=V1NFR2UD014) | 939 | 99% |
| N1 III (1-1-3 b) | 17 | MW603220 | *Psiloglonium* sp. | [KY378959](https://www.ncbi.nlm.nih.gov/nucleotide/1123883246?report=genbank&log$=nucltop&blast_rank=1&RID=V1NPSCAP016) | 941 | 99% |
| N1 III (1-2-2 a) | 18 | MW603221 | *Nemania bipapillata* | [GU292818](https://www.ncbi.nlm.nih.gov/nucleotide/296046133?report=genbank&log$=nucltop&blast_rank=1&RID=VEMBJFXW016) | 990 | 99% |
| N1 III (1-2-2 b) | 18b | MW603222 | *Nemania bipapillata* | [GU292818](https://www.ncbi.nlm.nih.gov/nucleotide/296046133?report=genbank&log$=nucltop&blast_rank=1&RID=VEMBJFXW016) | 990 | 99% |
| N1 III (1-3-1) | 20 | MW603223 | Fungal sp. | [KR012462](https://www.ncbi.nlm.nih.gov/nucleotide/928197966?report=genbank&log$=nucltop&blast_rank=1&RID=V1NY32TB016) | 1022 | 98% |
|  |  |  | *Pestalotiopsis neglecta* | [JX415485](https://www.ncbi.nlm.nih.gov/nucleotide/444302460?report=genbank&log$=nucltop&blast_rank=3&RID=V1NY32TB016) | 1014 | 99% |
| N1 III (1-2-1) | 22 | MW603224 | *Pestalotiopsis* sp. | [KC895524](https://www.ncbi.nlm.nih.gov/nucleotide/506954208?report=genbank&log$=nucltop&blast_rank=1&RID=V1URY01A016) | 1020 | 99% |
| N1 III (1-3-3) | 23 | MW603225 | Fungal sp. | [KU977555](https://www.ncbi.nlm.nih.gov/nucleotide/1152260421?report=genbank&log$=nucltop&blast_rank=1&RID=V1V3YZ3F014) | 664 | 99% |
|  |  |  | *Pestalotiopsis microspora* | [KU720061](https://www.ncbi.nlm.nih.gov/nucleotide/1060697748?report=genbank&log$=nucltop&blast_rank=2&RID=V1V3YZ3F014) | 664 | 99% |
| N1 III (2-1 a) | 24 | MW603226 | *Liberomyces* sp. | [KT336540](https://www.ncbi.nlm.nih.gov/nucleotide/KT336540.1?report=genbank&log$=nucltop&blast_rank=1&RID=5V9EKB6801R) | 935 | 99% |
| N1 III (2-1 b) | 25 | MW603227 | Fungal endophyte | [EU687056](https://www.ncbi.nlm.nih.gov/nucleotide/193246449?report=genbank&log$=nucltop&blast_rank=1&RID=VEM3VBWD014) | 832 | 94% |
|  |  |  | *Synnemellisia aurantia* | [KX866395](https://www.ncbi.nlm.nih.gov/nucleotide/1129819923?report=genbank&log$=nucltop&blast_rank=2&RID=VEM3VBWD014) | 800 | 93% |
| N1 III (2-1 c) | 26 | MW603228 | Fungal sp. strain | [KX098027](https://www.ncbi.nlm.nih.gov/nucleotide/1101019829?report=genbank&log$=nucltop&blast_rank=1&RID=VCC0K10701R) | 985 | 99% |
|  |  |  | *Lecanicillium sp.* | [KY683770](https://www.ncbi.nlm.nih.gov/nucleotide/1151053665?report=genbank&log$=nucltop&blast_rank=2&RID=VCC0K10701R) | 983 | 99% |
| N1 III (2-1 d) | 27 | MW603229 | Fungal sp. strain | [KY404948](https://www.ncbi.nlm.nih.gov/nucleotide/1128611668?report=genbank&log$=nucltop&blast_rank=1&RID=VCC6FFB701R) | 965 | 99% |
|  |  |  | *Nectria mauritiicola* | [HQ637271](https://www.ncbi.nlm.nih.gov/nucleotide/315200921?report=genbank&log$=nucltop&blast_rank=3&RID=VCC6FFB701R) | 965 | 99% |
| N1 III (2-1 e) | 28 | MW603230 | *Pleosporales* sp. | [HM992807](https://www.ncbi.nlm.nih.gov/nucleotide/304333829?report=genbank&log$=nucltop&blast_rank=1&RID=VCCCCVDR01R) | 867 | 99% |
|  |  |  | *Massarina* sp. | [AJ972794](https://www.ncbi.nlm.nih.gov/nucleotide/66990743?report=genbank&log$=nucltop&blast_rank=5&RID=VCCCCVDR01R) | 691 | 92% |
| N1 III (2-1 f) | 29 | MW603231 | Uncultured Ascomycota | [KJ194277](https://www.ncbi.nlm.nih.gov/nucleotide/KJ194277.1?report=genbank&log$=nucltop&blast_rank=1&RID=5V9MBNSS015) | 885 | 99% |
|  |  |  | Pleosporales sp. | [KJ867219](https://www.ncbi.nlm.nih.gov/nucleotide/KJ867219.1?report=genbank&log$=nucltop&blast_rank=3&RID=5V9MBNSS015) | 874 | 99% |
| N1 III (2-2 a) | 30 | MW603232 | *Xylaria* sp. | [JQ341076](https://www.ncbi.nlm.nih.gov/nucleotide/407378245?report=genbank&log$=nucltop&blast_rank=1&RID=V1G38UJM014) | 1020 | 99% |
| N1 III (2-2 b) | 31 | MW603233 | *Fusicolla violacea* | [JN198450](https://www.ncbi.nlm.nih.gov/nucleotide/339715435?report=genbank&log$=nucltop&blast_rank=1&RID=V1VJZ0PH014) | 918 | 98% |
| N1 III (2-3) | 32 | MW603234 | *Arthrinium phaeospermum* | [FJ462766](https://www.ncbi.nlm.nih.gov/nucleotide/217323599?report=genbank&log$=nucltop&blast_rank=1&RID=W6H960CW01R) | 1070 | 99% |
| N2 I (2-1 a) | 33 | MW603235 | Valsaceae sp. | [AB334109](https://www.ncbi.nlm.nih.gov/nucleotide/AB334109.1?report=genbank&log$=nucltop&blast_rank=1&RID=5VD2K2XJ015) | 791 | 93% |
| N2 I (2-1 c) | 35 | MW603236 | Marasmiaceae sp. | [KC771514](https://www.ncbi.nlm.nih.gov/nucleotide/KC771514.1?report=genbank&log$=nucltop&blast_rank=1&RID=5VFMM8PB015) | 1153 | 97% |
| N2 I (2-2) | 36 | MW603237 | *Trichoderma harzianum* | [KM278121](https://www.ncbi.nlm.nih.gov/nucleotide/748061305?report=genbank&log$=nucltop&blast_rank=1&RID=V1VVJ84F014) | 1072 | 99% |
| N2 I (2-3) | 37 | MW603238 | *Penicillium sumatrense* | [KT310939](https://www.ncbi.nlm.nih.gov/nucleotide/948273660?report=genbank&log$=nucltop&blast_rank=1&RID=VFANCUT701R) | 1387 | 99% |
| N2 II (1-1) | 38 | MW603239 | Xylariales sp. | [KX722242](https://www.ncbi.nlm.nih.gov/nucleotide/KX722242.1?report=genbank&log$=nucltop&blast_rank=1&RID=5VFZ7H95014) | 933 | 100% |
|  |  |  | *Nigrospora* sp. | [KT351614](https://www.ncbi.nlm.nih.gov/nucleotide/KT351614.1?report=genbank&log$=nucltop&blast_rank=4&RID=5VFZ7H95014) | 920 | 100% |
| N2 II (1-2) | 39 | MW603240 | *Pseudocercospora diplusodonii* | [NR147299](https://www.ncbi.nlm.nih.gov/nucleotide/1192789033?report=genbank&log$=nucltop&blast_rank=1&RID=VFAUNK9W01R) | 907 | 99% |
| N2 II (1-3 a) | 40 | MW603241 | *Biscogniauxia* sp. | [KP306931](https://www.ncbi.nlm.nih.gov/nucleotide/KP306931.1?report=genbank&log$=nucltop&blast_rank=1&RID=5VJ9NGXT01R) | 1171 | 99% |
| N2 II (1-3 b) | 41 | MW603242 | *Trichoderma* sp. | [KX357833](https://www.ncbi.nlm.nih.gov/nucleotide/1139736428?report=genbank&log$=nucltop&blast_rank=1&RID=VFAYBN7101R) | 1057 | 99% |
| N2 II (1-3 c) | 42 | MW603243 | *Cyphellophora europaea* | [LN827691](https://www.ncbi.nlm.nih.gov/nucleotide/765364793?report=genbank&log$=nucltop&blast_rank=1&RID=VFB1DV8201R) | 617 | 86% |
| N2 II (1-3 d) | 43 | MW603244 | Xylariaceae sp. | [AB741601](https://www.ncbi.nlm.nih.gov/nucleotide/AB741601.1?report=genbank&log$=nucltop&blast_rank=1&RID=B9DYNNSB015) | 1781 | 98% |
|  |  |  | *Annulohypoxylon nitens* | [KU684021](https://www.ncbi.nlm.nih.gov/nucleotide/KU684021.1?report=genbank&log$=nucltop&blast_rank=2&RID=B9DYNNSB015) | 1768 | 98% |
| N2 II (2-2 a) | 44 | MW603245 | Agaricales sp. | [KP006344](https://www.ncbi.nlm.nih.gov/nucleotide/KP006344.1?report=genbank&log$=nucltop&blast_rank=1&RID=DXDTJV56014) | 497 | 83% |
|  |  |  | Marasmiaceae sp. | [KC771514](https://www.ncbi.nlm.nih.gov/nucleotide/KC771514.1?report=genbank&log$=nucltop&blast_rank=2&RID=DXDTJV56014) | 490 | 81% |
| N2 II (2-2 b) | 45 | MW603246 | Fungal endophyte | [KF673722](https://www.ncbi.nlm.nih.gov/nucleotide/555636237?report=genbank&log$=nucltop&blast_rank=1&RID=VFB5ZUSJ01R) | 1013 | 99% |
|  |  |  | *Penicillium kloeckeri* | [HM469393](https://www.ncbi.nlm.nih.gov/nucleotide/326579915?report=genbank&log$=nucltop&blast_rank=11&RID=VFB5ZUSJ01R) | 1013 | 99% |
| N2 II (2-2 c) | 46 | MW603247 | *Cylindrocladium* sp. | [KP972552](https://www.ncbi.nlm.nih.gov/nucleotide/829580571?report=genbank&log$=nucltop&blast_rank=1&RID=VFBBYMNN01R) | 957 | 99% |
| N2 II (2-3 a) | 47 | MW603248 | Marasmiaceae sp. | [KC771514](https://www.ncbi.nlm.nih.gov/nucleotide/KC771514.1?report=genbank&log$=nucltop&blast_rank=1&RID=DXEBZSA9014) | 399 | 84% |
|  |  |  | Uncultured *Marasmius* | [KF718232](https://www.ncbi.nlm.nih.gov/nucleotide/KF718232.1?report=genbank&log$=nucltop&blast_rank=4&RID=DXEBZSA9014) | 283 | 80% |
| N2 II (2-3 b) | 48 | MW603249 | *Cyphellophora europaea* | [LN827691](https://www.ncbi.nlm.nih.gov/nucleotide/765364793?report=genbank&log$=nucltop&blast_rank=1&RID=V1XSC34V014) | 616 | 86% |
| N2 III (1-1 a) | 49 | MW603250 | Pleosporales sp. | [HQ914897](https://www.ncbi.nlm.nih.gov/nucleotide/327387929?report=genbank&log$=nucltop&blast_rank=1&RID=VFBG865D01R) | 894 | 99% |
|  |  |  | *Nigrograna mackinnonii* | [KC288117](https://www.ncbi.nlm.nih.gov/nucleotide/508078114?report=genbank&log$=nucltop&blast_rank=8&RID=VFBG865D01R) | 865 | 98% |
| N2 III (1-1 b) | 50 | MW603251 | *Xylaria* sp. | [KM513587](https://www.ncbi.nlm.nih.gov/nucleotide/KM513587.1?report=genbank&log$=nucltop&blast_rank=1&RID=5VNW7K0M015) | 989 | 99% |
| N2 III (1-1 c) | 51 | MW603252 | Marasmiaceae sp. | [KC771514](https://www.ncbi.nlm.nih.gov/nucleotide/KC771514.1?report=genbank&log$=nucltop&blast_rank=1&RID=DX6RM6D1014) | 1027 | 94% |
|  |  |  | *Marasmiellus candidus* | [EF175516](https://www.ncbi.nlm.nih.gov/nucleotide/EF175516.1?report=genbank&log$=nucltop&blast_rank=2&RID=DX6RM6D1014) | 817 | 88% |
| N2 III (1-1 d) | 52 | MW603253 | *Trichoderma harzianum* | [KC113293](https://www.ncbi.nlm.nih.gov/nucleotide/443682267?report=genbank&log$=nucltop&blast_rank=1&RID=V1Y0ZCTR014) | 1077 | 99% |
| N2 III (1-1 e) | 53 | MW603254 | *Trichoderma* cfr. *harzianum* | [MF109004](https://www.ncbi.nlm.nih.gov/nucleotide/1193115770?report=genbank&log$=nucltop&blast_rank=1&RID=V1Y75W9H016) | 1202 | 100% |
| N2 III (1-2) | 54 | MW603255 | *Arthrinium marii* | [KF144901](https://www.ncbi.nlm.nih.gov/nucleotide/511343100?report=genbank&log$=nucltop&blast_rank=1&RID=V1YDEUYR016) | 1070 | 99% |
| N2 III (1-3 a) | 55 | MW603256 | *Trichoderma* sp. | [KX357833](https://www.ncbi.nlm.nih.gov/nucleotide/1139736428?report=genbank&log$=nucltop&blast_rank=1&RID=V1YMT1GY016) | 1062 | 99% |
| N2 III (1-3 b) | 56 | MW603257 | *Annulohypoxylon nitens* | [KX722230](https://www.ncbi.nlm.nih.gov/nucleotide/1151248780?report=genbank&log$=nucltop&blast_rank=1&RID=VCCVNV6G01R) | 955 | 99% |
| N2 III (1-3 c) | 57 | MW603258 | Marasmiaceae sp. | [KC771514](https://www.ncbi.nlm.nih.gov/nucleotide/KC771514.1?report=genbank&log$=nucltop&blast_rank=1&RID=DX747PCE015) | 944 | 91% |
|  |  |  | *Marasmiellus candidus* | [EF175516](https://www.ncbi.nlm.nih.gov/nucleotide/EF175516.1?report=genbank&log$=nucltop&blast_rank=2&RID=DX747PCE015) | 723 | 86% |
| N2 III (1-3 d) | 58 | MW603259 | Marasmiaceae sp. | [KC771514](https://www.ncbi.nlm.nih.gov/nucleotide/KC771514.1?report=genbank&log$=nucltop&blast_rank=1&RID=DX7PRKKY014) | 931 | 91% |
|  |  |  | *Marasmiellus candidus* | [EF175516](https://www.ncbi.nlm.nih.gov/nucleotide/EF175516.1?report=genbank&log$=nucltop&blast_rank=2&RID=DX7PRKKY014) | 739 | 86% |
| N2 III (2-1 a) | 59 | MW603260 | Marasmiaceae sp. | [KC771514](https://www.ncbi.nlm.nih.gov/nucleotide/482679691?report=genbank&log$=nucltop&blast_rank=1&RID=V1Z1A1FH014) | 1166 | 97% |
| N2 III (2-1 b) | 60 | MW603261 | Marasmiaceae sp. | [KC771514](https://www.ncbi.nlm.nih.gov/nucleotide/482679691?report=genbank&log$=nucltop&blast_rank=1&RID=V1Z7058X014) | 1166 | 97% |
| N2 III (2-1 c) | 61 | MW603262 | Dothideomycetes sp. | [JQ760421](https://www.ncbi.nlm.nih.gov/nucleotide/387353283?report=genbank&log$=nucltop&blast_rank=1&RID=VFBPKW4P01R) | 1098 | 99% |
|  |  |  | *Ochroconis humicola* | [HQ608103](https://www.ncbi.nlm.nih.gov/nucleotide/312434581?report=genbank&log$=nucltop&blast_rank=4&RID=VFBPKW4P01R) | 1083 | 99% |
| N2 III (2-2 a) | 62 | MW603263 | *Lecanicillium* sp. | [LT598646](https://www.ncbi.nlm.nih.gov/nucleotide/1043260257?report=genbank&log$=nucltop&blast_rank=1&RID=W6HM2EYC01R) | 992 | 99% |
| N2 III (2-2 b) | 63 | MW603264 | Uncultured fungus | [FJ528708](https://www.ncbi.nlm.nih.gov/nucleotide/257122681?report=genbank&log$=nucltop&blast_rank=1&RID=VCD0E5KR01R) | 1048 | 99% |
|  |  |  | *Deconica coprophila* | [KJ780773](https://www.ncbi.nlm.nih.gov/nucleotide/667673676?report=genbank&log$=nucltop&blast_rank=2&RID=VCD0E5KR01R) | 1022 | 99% |
| N2 III (2-2 c) | 64 | MW603265 | Marasmiaceae sp. | [KC771514](https://www.ncbi.nlm.nih.gov/nucleotide/KC771514.1?report=genbank&log$=nucltop&blast_rank=1&RID=DX7XVPAV015) | 848 | 89% |
|  |  |  | *Marasmiellus candidus* | [EF175516](https://www.ncbi.nlm.nih.gov/nucleotide/EF175516.1?report=genbank&log$=nucltop&blast_rank=2&RID=DX7XVPAV015) | 675 | 84% |
| N2 III (2-3) | 65 | MW603266 | Marasmiaceae sp. | [KC771514](https://www.ncbi.nlm.nih.gov/nucleotide/KC771514.1?report=genbank&log$=nucltop&blast_rank=1&RID=DX89K5YD014) | 931 | 91% |
|  |  |  | *Marasmiellus candidus* | [EF175516](https://www.ncbi.nlm.nih.gov/nucleotide/EF175516.1?report=genbank&log$=nucltop&blast_rank=2&RID=DX89K5YD014) | 728 | 86% |
| N3 I (1-3 b) | 68 | MW603267 | Sordariomycetes sp. | [JX174124](https://www.ncbi.nlm.nih.gov/nucleotide/JX174124.1?report=genbank&log$=nucltop&blast_rank=1&RID=5Y1DS0PF015) | 922 | 98% |
|  |  |  | *Fusicolla violacea* | [JN198450](https://www.ncbi.nlm.nih.gov/nucleotide/JN198450.1?report=genbank&log$=nucltop&blast_rank=2&RID=5Y1DS0PF015) | 918 | 99% |
| N3 I (2-1 a) | 69 | MW603268 | Uncultured fungus | [MF976788](https://www.ncbi.nlm.nih.gov/nucleotide/MF976788.1?report=genbank&log$=nucltop&blast_rank=1&RID=5Y1R4669015) | 606 | 90% |
| N3 I (2-1 b) | 70 | MW603269 | *Hypoxylon munkii* | [JN979436](https://www.ncbi.nlm.nih.gov/nucleotide/JN979436.1?report=genbank&log$=nucltop&blast_rank=1&RID=5Y1YAREP014) | 1002 | 97% |
| N3 I (2-1 c) | 71 | MW603270 | *Cylindrocladium sp.* | [KP972552](https://www.ncbi.nlm.nih.gov/nucleotide/829580571?report=genbank&log$=nucltop&blast_rank=1&RID=VFBYT6WV01R) | 961 | 99% |
| N3 I (2-2 a) | 72 | MW603271 | *Cyphellophora europaea* | [LN827691](https://www.ncbi.nlm.nih.gov/nucleotide/765364793?report=genbank&log$=nucltop&blast_rank=1&RID=VCDF12XS01R) | 623 | 87% |
| N3 I (2-2 b) | 73 | MW603272 | Uncultured fungus | [KT328903](https://www.ncbi.nlm.nih.gov/nucleotide/KT328903.1?report=genbank&log$=nucltop&blast_rank=1&RID=5Y22GEK7014) | 647 | 88% |
|  |  |  | *Sarcinomyces* sp. | [KM056297](https://www.ncbi.nlm.nih.gov/nucleotide/KM056297.1?report=genbank&log$=nucltop&blast_rank=4&RID=5Y22GEK7014) | 640 | 91% |
| N3 I (2-3) | 74 | MW603273 | *Biscogniauxia capnodes* | [EF026131](https://www.ncbi.nlm.nih.gov/nucleotide/122935543?report=genbank&log$=nucltop&blast_rank=1&RID=W6HVUB3P01R) | 1258 | 97% |
| N3 II (2-1 a) | 75 | MW603274 | *Biatriospora* sp. | [MF588874](https://www.ncbi.nlm.nih.gov/nucleotide/MF588874.1?report=genbank&log$=nucltop&blast_rank=1&RID=5Y29TRGH014) | 902 | 99% |
| N3 II (2-1 b) | 76 | MW603275 | Fungal sp. strain | [KR004881](https://www.ncbi.nlm.nih.gov/nucleotide/940816734?report=genbank&log$=nucltop&blast_rank=1&RID=VCDNTUPS01R) | 989 | 99% |
|  |  |  | *Nectria mauritiicola* | [HQ637271](https://www.ncbi.nlm.nih.gov/nucleotide/315200921?report=genbank&log$=nucltop&blast_rank=2&RID=VCDNTUPS01R) | 985 | 99% |
| N3 II (2-2 b) | 78 | MW603276 | Fungal sp. | [FJ025163](https://www.ncbi.nlm.nih.gov/nucleotide/205277714?report=genbank&log$=nucltop&blast_rank=1&RID=VCDVW1XU01R) | 970 | 99% |
|  |  |  | *Clonostachys rosea* | [KU350706](https://www.ncbi.nlm.nih.gov/nucleotide/1026943344?report=genbank&log$=nucltop&blast_rank=2&RID=VCDVW1XU01R) | 965 | 99% |
| N3 II (2-3 a) | 79 | MW603277 | Sordariomycetes sp. | [JX174124](https://www.ncbi.nlm.nih.gov/nucleotide/JX174124.1?report=genbank&log$=nucltop&blast_rank=1&RID=60H9Z2CS013) | 922 | 98% |
|  |  |  | *Fusicolla violacea* | [JN198450](https://www.ncbi.nlm.nih.gov/nucleotide/JN198450.1?report=genbank&log$=nucltop&blast_rank=2&RID=60H9Z2CS013) | 918 | 99% |
| N3 II (2-3 b) | 80 | MW603278 | *Fusicolla violacea* | [JN198450](https://www.ncbi.nlm.nih.gov/nucleotide/JN198450.1?report=genbank&log$=nucltop&blast_rank=1&RID=AYV936F3014) | 935 | 99% |
| N3 II (2-3 c) | 81 | MW603279 | *Geosmithia* sp. | [KF934490](https://www.ncbi.nlm.nih.gov/nucleotide/567849968?report=genbank&log$=nucltop&blast_rank=1&RID=VCE58DCB01R) | 527 | 87% |
| N3 II (2-3 d) | 82 | MW603280 | *Fusicolla violacea* | [JN198450](https://www.ncbi.nlm.nih.gov/nucleotide/339715435?report=genbank&log$=nucltop&blast_rank=1&RID=VCF8EXG1014) | 926 | 98% |
| N3 III (1-2) | 83 | MW603281 | *Pestalotiopsis vismiae* | [KP689172](https://www.ncbi.nlm.nih.gov/nucleotide/755984218?report=genbank&log$=nucltop&blast_rank=1&RID=VCFCNSEY016) | 1042 | 99% |
| N3 III (2-1) | 84 | MW603282 | Fungal sp. strain | [KU728368](https://www.ncbi.nlm.nih.gov/nucleotide/1047835710?report=genbank&log$=nucltop&blast_rank=1&RID=V21N6MF1016) | 942 | 99% |
|  |  |  | *Cladosporium cladosporioides* | [HQ671181](https://www.ncbi.nlm.nih.gov/nucleotide/317574101?report=genbank&log$=nucltop&blast_rank=2&RID=V21N6MF1016) | 941 | 99% |
| N3 III (2-2) | 85 | MW603283 | *Xylaria grammica* | [JQ862684](https://www.ncbi.nlm.nih.gov/nucleotide/429888630?report=genbank&log$=nucltop&blast_rank=1&RID=V220CYF3014) | 1000 | 99% |
| N3 III (2-3 a) | 86 | MW603284 | *Hypoxylon* sp. | [JQ862706](https://www.ncbi.nlm.nih.gov/nucleotide/429888652?report=genbank&log$=nucltop&blast_rank=1&RID=V226ARXV014) | 1024 | 99% |
| N3 III (2-3 b) | 87 | MW603285 | *Cladophialophora* sp. | [EU139132](https://www.ncbi.nlm.nih.gov/nucleotide/160552295?report=genbank&log$=nucltop&blast_rank=1&RID=VCFKTJKR014) | 819 | 92% |
| N4 I (1-1) | 89 | MW603286 | *Trichoderma gamsii* | [EF488141](https://www.ncbi.nlm.nih.gov/nucleotide/152937909?report=genbank&log$=nucltop&blast_rank=1&RID=VCGWG3HZ014) | 1040 | 99% |
| N4 I (1-2 a) | 90 | MW603287 | *Coniothyrium nitidae* | [GU355659](https://www.ncbi.nlm.nih.gov/nucleotide/296042322?report=genbank&log$=nucltop&blast_rank=1&RID=VCH2ZX8Y014) | 957 | 99% |
| N4 I (1-2 b) | 91 | MW603288 | *Coniothyrium nitidae* | [GU355659](https://www.ncbi.nlm.nih.gov/nucleotide/296042322?report=genbank&log$=nucltop&blast_rank=1&RID=VCH6XTPC016) | 957 | 99% |
| N4 I (1-3) | 92 | MW603289 | Hypocreales sp. | [KP322765](https://www.ncbi.nlm.nih.gov/nucleotide/757958769?report=genbank&log$=nucltop&blast_rank=1&RID=VCHCBU47014) | 965 | 98% |
|  |  |  | *Spicellum* sp. | [GU183172](https://www.ncbi.nlm.nih.gov/nucleotide/270303629?report=genbank&log$=nucltop&blast_rank=6&RID=VCHCBU47014) | 869 | 95% |
| N4 I (2-1 a) | 93 | MW603290 | Uncultured *Cladosporium* | [HQ588319](https://www.ncbi.nlm.nih.gov/nucleotide/315139375?report=genbank&log$=nucltop&blast_rank=1&RID=VCHK0F61014) | 937 | 99% |
| N4 I (2-1 b) | 94 | MW603291 | *Alternaria alternata* | [KJ173524](https://www.ncbi.nlm.nih.gov/nucleotide/608605141?report=genbank&log$=nucltop&blast_rank=1&RID=VCHTD4XC016) | 981 | 99% |
| N4 I (2-1 c) | 95 | MW603292 | Fungal sp. | [KU977695](https://www.ncbi.nlm.nih.gov/nucleotide/KU977695.1?report=genbank&log$=nucltop&blast_rank=1&RID=AYVFF466014) | 893 | 97% |
|  |  |  | *Paraconiothyrium* sp. | [KY488353](https://www.ncbi.nlm.nih.gov/nucleotide/KY488353.1?report=genbank&log$=nucltop&blast_rank=6&RID=AYVFF466014) | 832 | 95% |
| N4 I (2-2 a) | 96 | MW603293 | *Cyphellophora europaea* | [LN827691](https://www.ncbi.nlm.nih.gov/nucleotide/765364793?report=genbank&log$=nucltop&blast_rank=1&RID=VCHXTU8E016) | 614 | 86% |
| N4 I (2-2 b) | 97 | MW603294 | *Clonostachys rosea* | [KJ540101](https://www.ncbi.nlm.nih.gov/nucleotide/613845931?report=genbank&log$=nucltop&blast_rank=1&RID=VCJ0TA24016) | 961 | 99% |
| N4 I (2-2 c) | 98 | MW603295 | Fungal sp. | [GQ996096](https://www.ncbi.nlm.nih.gov/nucleotide/GQ996096.1?report=genbank&log$=nucltop&blast_rank=1&RID=AYVV4KBT014) | 678 | 93% |
|  |  |  | *Fusarium decemcellulare* | [KY024396](https://www.ncbi.nlm.nih.gov/nucleotide/KY024396.1?report=genbank&log$=nucltop&blast_rank=9&RID=AYVV4KBT014) | 566 | 91% |
| N4 I (2-2 d) | 99 | MW603296 | *Cyphellophora europaea* | [LN827691](https://www.ncbi.nlm.nih.gov/nucleotide/765364793?report=genbank&log$=nucltop&blast_rank=1&RID=V22GG0B6014) | 621 | 87% |
| N4 I (2-2 e) | 100 | MW603297 | *Cyphellophora europaea* | [LN827691](https://www.ncbi.nlm.nih.gov/nucleotide/765364793?report=genbank&log$=nucltop&blast_rank=1&RID=V22PXU1A014) | 621 | 87% |
| N4 I (2-3 a) | 101 | MW603298 | *Cyphellophora europaea* | [LN827691](https://www.ncbi.nlm.nih.gov/nucleotide/765364793?report=genbank&log$=nucltop&blast_rank=1&RID=V22VP9RX016) | 619 | 87% |
| N4 I (2-3 b) | 102 | MW603299 | Hypocreales sp. | [JQ411375](https://www.ncbi.nlm.nih.gov/nucleotide/JQ411375.1?report=genbank&log$=nucltop&blast_rank=1&RID=AYW1G44D015) | 815 | 94% |
|  |  |  | *Tolypocladium inflatum* | [JF796050](https://www.ncbi.nlm.nih.gov/nucleotide/JF796050.1?report=genbank&log$=nucltop&blast_rank=2&RID=AYW1G44D015) | 808 | 94% |
| N4 II (1-1) | 103 | MW603300 | *Letendraea helminthicola* | [KJ774052](https://www.ncbi.nlm.nih.gov/nucleotide/667673641?report=genbank&log$=nucltop&blast_rank=1&RID=VCJ73B2C014) | 1235 | 99% |
| N4 II (1-2) | 104 | MW603301 | *Coniothyrium nitidae* | [GU355659](https://www.ncbi.nlm.nih.gov/nucleotide/296042322?report=genbank&log$=nucltop&blast_rank=1&RID=V23CM09X014) | 959 | 99% |
| N4 II (1-3) | 105 | MW603302 | Sordariomycetes sp. | [JX174143](https://www.ncbi.nlm.nih.gov/nucleotide/403314327?report=genbank&log$=nucltop&blast_rank=1&RID=V24JYCMH014) | 920 | 98% |
|  |  |  | *Bionectria* sp. | [HQ022506](https://www.ncbi.nlm.nih.gov/nucleotide/307239271?report=genbank&log$=nucltop&blast_rank=2&RID=V24JYCMH014) | 865 | 98% |
| N4 II (2-1 a) | 106 | MW603303 | *Cladosporium cucumerinum* | [KF986443](https://www.ncbi.nlm.nih.gov/nucleotide/KF986443.1?report=genbank&log$=nucltop&blast_rank=1&RID=AYW70HKA015) | 1010 | 99% |
| N4 II (2-1 b) | 107 | MW603304 | *Veronaea* sp. | [HM992819](https://www.ncbi.nlm.nih.gov/nucleotide/304333841?report=genbank&log$=nucltop&blast_rank=1&RID=VCJBSHTK014) | 985 | 97% |
| N4 II (2-2) | 108 | MW603305 | Eurotiomycetes sp. | [KX909058](https://www.ncbi.nlm.nih.gov/nucleotide/1103850227?report=genbank&log$=nucltop&blast_rank=1&RID=VFC2STVJ01R) | 987 | 99% |
|  |  |  | *Talaromyces funiculosus* | [GU980968](https://www.ncbi.nlm.nih.gov/nucleotide/294847839?report=genbank&log$=nucltop&blast_rank=4&RID=VFC2STVJ01R) | 981 | 99% |
| N4 II (2-3 a) | 109 | MW603306 | *Cyphellophora europaea* | [LN827691](https://www.ncbi.nlm.nih.gov/nucleotide/765364793?report=genbank&log$=nucltop&blast_rank=1&RID=VCK201M701R) | 630 | 87% |
| N4 II (2-3 b) | 110 | MW603307 | Uncultured fungus | [KF800273](https://www.ncbi.nlm.nih.gov/nucleotide/571431167?report=genbank&log$=nucltop&blast_rank=1&RID=VCK7H9UG01R) | 977 | 99% |
|  |  |  | *Lasionectria* sp. | [JX306092](https://www.ncbi.nlm.nih.gov/nucleotide/404435317?report=genbank&log$=nucltop&blast_rank=2&RID=VCK7H9UG01R) | 972 | 99% |
| N4 II (2-3 c) | 111 | MW603308 | Uncultured fungus | [JN890292](https://www.ncbi.nlm.nih.gov/nucleotide/383290653?report=genbank&log$=nucltop&blast_rank=1&RID=VCKFWR5V01R) | 1022 | 99% |
|  |  |  | *Cyphellophora guyanensis* | [GU225943](https://www.ncbi.nlm.nih.gov/nucleotide/299835226?report=genbank&log$=nucltop&blast_rank=3&RID=VCKFWR5V01R) | 1007 | 98% |
| N4 III (1-1) | 112 | MW603309 | *Coniothyrium nitidae* | [GU355659](https://www.ncbi.nlm.nih.gov/nucleotide/296042322?report=genbank&log$=nucltop&blast_rank=1&RID=VCKXKYCT016) | 959 | 99% |
| N4 III (1-2 a) | 113 | MW603310 | *Clonostachys rosea* | [KU350706](https://www.ncbi.nlm.nih.gov/nucleotide/1026943344?report=genbank&log$=nucltop&blast_rank=1&RID=V47KP1WB014) | 970 | 99% |
| N4 III (1-2 b) | 114 | MW603311 | Uncultured Basidiomycota | [GU328605](https://www.ncbi.nlm.nih.gov/nucleotide/GU328605.1?report=genbank&log$=nucltop&blast_rank=1&RID=AYWCGB0N014) | 510 | 84% |
|  |  |  | *Peniophora* sp. | [KC176330](https://www.ncbi.nlm.nih.gov/nucleotide/KC176330.1?report=genbank&log$=nucltop&blast_rank=5&RID=AYWCGB0N014) | 414 | 84% |
| N4 III (1-2 c) | 115 | MW603312 | *Cyphellophora europaea* | [LN827691](https://www.ncbi.nlm.nih.gov/nucleotide/765364793?report=genbank&log$=nucltop&blast_rank=1&RID=V47TSDZ9014) | 621 | 86% |
| N4 III (1-2 d) | 116 | MW603313 | Fungal sp. | [GQ996096](https://www.ncbi.nlm.nih.gov/nucleotide/301068718?report=genbank&log$=nucltop&blast_rank=1&RID=V481J3HC016) | 667 | 92% |
|  |  |  | *Pseudonectria* sp. | [JQ425377](https://www.ncbi.nlm.nih.gov/nucleotide/385258040?report=genbank&log$=nucltop&blast_rank=2&RID=V481J3HC016) | 560 | 91% |
| N4 III (1-2 e) | 117 | MW603314 | Uncultured *Penicillium* | [FN394532](https://www.ncbi.nlm.nih.gov/nucleotide/238805279?report=genbank&log$=nucltop&blast_rank=1&RID=VCMKMW4V01R) | 994 | 99% |
| N4 III (1-2 f) | 118 | MW603315 | Uncultured fungus | [JN890292](https://www.ncbi.nlm.nih.gov/nucleotide/383290653?report=genbank&log$=nucltop&blast_rank=1&RID=V488JTTV016) | 1037 | 99% |
|  |  |  | *Cyphellophora eucalypti* | [GQ303274](https://www.ncbi.nlm.nih.gov/nucleotide/258578849?report=genbank&log$=nucltop&blast_rank=3&RID=V488JTTV016) | 1020 | 99% |
| N4 III (1-3) | 119 | MW603316 | *Coprinellus disseminatus* | [JQ922135](https://www.ncbi.nlm.nih.gov/nucleotide/511190123?report=genbank&log$=nucltop&blast_rank=1&RID=VCN450ND01R) | 1201 | 99% |
| N4 III (2-1 a) | 120 | MW603317 | *Fusicolla violacea* | [JN198450](https://www.ncbi.nlm.nih.gov/nucleotide/339715435?report=genbank&log$=nucltop&blast_rank=1&RID=VCN9NFX001R) | 911 | 99% |
| N4 III (2-1 b) | 121 | MW603318 | *Veronaea japonica* | [KX302057](https://www.ncbi.nlm.nih.gov/nucleotide/1139417840?report=genbank&log$=nucltop&blast_rank=1&RID=V48KRJFM014) | 941 | 95% |
| N4 III (2-3) | 123 | MW603319 | Uncultured Basidiomycota | [GU328605](https://www.ncbi.nlm.nih.gov/nucleotide/GU328605.1?report=genbank&log$=nucltop&blast_rank=1&RID=AYWJW3A7014) | 508 | 84% |
|  |  |  | *Peniophora* sp. | [KC176330](https://www.ncbi.nlm.nih.gov/nucleotide/KC176330.1?report=genbank&log$=nucltop&blast_rank=5&RID=AYWJW3A7014) | 412 | 84% |
| P1 I (2-1) | 124 | MW603320 | Uncultured fungus | [GQ999239](https://www.ncbi.nlm.nih.gov/nucleotide/299767647?report=genbank&log$=nucltop&blast_rank=1&RID=VHVZ5WER01R) | 968 | 99% |
|  |  |  | *Penicillium wollemiicola* | [KJ174314](https://www.ncbi.nlm.nih.gov/nucleotide/651277165?report=genbank&log$=nucltop&blast_rank=2&RID=VHVZ5WER01R) | 865 | 96% |
| P1 I (2-2) | 125 | MW603321 | *Fusicolla violacea* | [JN198450](https://www.ncbi.nlm.nih.gov/nucleotide/339715435?report=genbank&log$=nucltop&blast_rank=1&RID=VCNTUMMZ01R) | 920 | 98% |
| P1 I (2-3) | 126 | MW603322 | *Trichoderma harzianum* | [KC874893](https://www.ncbi.nlm.nih.gov/nucleotide/589919715?report=genbank&log$=nucltop&blast_rank=1&RID=WH0T6PK4015) | 1062 | 99% |
| P1 II (2-1) | 127 | MW603323 | *Cladosporium* sp. | [KF293973](https://www.ncbi.nlm.nih.gov/nucleotide/KF293973.1?report=genbank&log$=nucltop&blast_rank=1&RID=AYYBMG4B014) | 937 | 99% |
| P1 II (2-2) | 128 | MW603324 | Uncultured fungus | [GQ999239](https://www.ncbi.nlm.nih.gov/nucleotide/299767647?report=genbank&log$=nucltop&blast_rank=1&RID=VHWAF1C301R) | 974 | 99% |
|  |  |  | *Penicillium wollemiicola* | [KJ174314](https://www.ncbi.nlm.nih.gov/nucleotide/651277165?report=genbank&log$=nucltop&blast_rank=2&RID=VHWAF1C301R) | 872 | 96% |
| P1 II (2-3) | 130 | MW603325 | Uncultured endophytic fungus | [LT560081](https://www.ncbi.nlm.nih.gov/nucleotide/1196823827?report=genbank&log$=nucltop&blast_rank=1&RID=V48VSUUU016) | 455 | 100% |
|  |  |  | *Purpureocillium lilacinum* | [KX347471](https://www.ncbi.nlm.nih.gov/nucleotide/1087919465?report=genbank&log$=nucltop&blast_rank=2&RID=V48VSUUU016) | 455 | 100% |
| P1 III (2-1-1) | 131 | MW603326 | *Fusicolla violacea* | [JN198450](https://www.ncbi.nlm.nih.gov/nucleotide/339715435?report=genbank&log$=nucltop&blast_rank=1&RID=V492VHZS016) | 915 | 98% |
| P1 III (2-1-2) | 132 | MW603327 | *Pestalotiopsis oxyanthi* | [KP900246](https://www.ncbi.nlm.nih.gov/nucleotide/829098570?report=genbank&log$=nucltop&blast_rank=1&RID=V498JCRK014) | 931 | 99% |
| P1 III (2-2) | 133 | MW603328 | *Fusicolla violacea* | [JN198450](https://www.ncbi.nlm.nih.gov/nucleotide/339715435?report=genbank&log$=nucltop&blast_rank=1&RID=V49C56V6014) | 920 | 98% |
| P1 III (2-3) | 134 | MW603329 | *Verticillium* cfr. *aranearum* | [AF108467](https://www.ncbi.nlm.nih.gov/nucleotide/4836219?report=genbank&log$=nucltop&blast_rank=1&RID=V49G8E32014) | 1009 | 99% |
| P2 I (2-1-1 a) | 135 | MW603330 | Fungal sp. | [GQ996176](https://www.ncbi.nlm.nih.gov/nucleotide/301068798?report=genbank&log$=nucltop&blast_rank=1&RID=V49RZWF4014) | 878 | 98% |
|  |  |  | *Acremonium variecolor* | [LN714516](https://www.ncbi.nlm.nih.gov/nucleotide/735997125?report=genbank&log$=nucltop&blast_rank=2&RID=V49RZWF4014) | 874 | 99% |
| P2 I (2-1-1 b) | 136 | MW603331 | Uncultured fungus | [GQ999239](https://www.ncbi.nlm.nih.gov/nucleotide/299767647?report=genbank&log$=nucltop&blast_rank=1&RID=VHWNDTSC01R) | 968 | 99% |
|  |  |  | *Penicillium wollemiicola* | [KJ174314](https://www.ncbi.nlm.nih.gov/nucleotide/651277165?report=genbank&log$=nucltop&blast_rank=2&RID=VHWNDTSC01R) | 867 | 96% |
| P2 I (2-1-2) | 137 | MW603332 | *Talaromyces verruculosus* | [KX258799](https://www.ncbi.nlm.nih.gov/nucleotide/1050213718?report=genbank&log$=nucltop&blast_rank=1&RID=VCNYGHDX01R) | 941 | 98% |
| P2 I (2-1) | 138 | MW603333 | Fungal endophyte | [JQ846051](https://www.ncbi.nlm.nih.gov/nucleotide/387773601?report=genbank&log$=nucltop&blast_rank=1&RID=VCP4AZ7301R) | 824 | 96% |
|  |  |  | *Setophoma* sp. | [KP050652](https://www.ncbi.nlm.nih.gov/nucleotide/742524453?report=genbank&log$=nucltop&blast_rank=12&RID=VCP4AZ7301R) | 797 | 96% |
| P2 I (2-3) | 139 | MW603334 | *Cladosporium uredinicola* | [KM513616](https://www.ncbi.nlm.nih.gov/nucleotide/742522529?report=genbank&log$=nucltop&blast_rank=1&RID=VCPNWK60014) | 950 | 99% |
| P2 II (2-2-1) | 140 | MW603335 | *Cladosporium cladosporioides* | [KY114882](https://www.ncbi.nlm.nih.gov/nucleotide/1148303064?report=genbank&log$=nucltop&blast_rank=1&RID=VCPUB1VN014) | 948 | 99% |
| P2 II (2-2-2) | 141 | MW603336 | *Penicillium meleagrinum* var. *viridiflavum* | [KT310976](https://www.ncbi.nlm.nih.gov/nucleotide/948273697?report=genbank&log$=nucltop&blast_rank=1&RID=VHX25F6X01R) | 1018 | 99% |
| P2 II (2-2-3) | 142 | MW603337 | *Trichoderma afroharzianum* | [KX357846](https://www.ncbi.nlm.nih.gov/nucleotide/1139736441?report=genbank&log$=nucltop&blast_rank=1&RID=VCPX5XKW014) | 1068 | 99% |
| P2 II (2-3-1) | 143 | MW603338 | Uncultured fungus | [GQ999239](https://www.ncbi.nlm.nih.gov/nucleotide/299767647?report=genbank&log$=nucltop&blast_rank=1&RID=VHX7XEHU01R) | 974 | 99% |
|  |  |  | *Penicillium wollemiicola* | [KJ174314](https://www.ncbi.nlm.nih.gov/nucleotide/651277165?report=genbank&log$=nucltop&blast_rank=2&RID=VHX7XEHU01R) | 872 | 96% |
| P2 II (2-3-2) | 144 | MW603339 | *Colletotrichum gloeosporioides* | [AB470881](https://www.ncbi.nlm.nih.gov/nucleotide/237769574?report=genbank&log$=nucltop&blast_rank=1&RID=VCR0MHND016) | 937 | 96% |
| P2 III (2-1) | 145 | MW603340 | *Fusicolla violacea* | [JN198450](https://www.ncbi.nlm.nih.gov/nucleotide/339715435?report=genbank&log$=nucltop&blast_rank=1&RID=VCR631CZ014) | 926 | 98% |
| P2 III (2-3 a) | 146 | MW603341 | Sordariomycetes sp. | [JX174124](https://www.ncbi.nlm.nih.gov/nucleotide/403314308?report=genbank&log$=nucltop&blast_rank=1&RID=VCRFA32C016) | 911 | 98% |
|  |  |  | *Fusicolla violacea* | [JN198450](https://www.ncbi.nlm.nih.gov/nucleotide/339715435?report=genbank&log$=nucltop&blast_rank=2&RID=VCRFA32C016) | 909 | 98% |
| P2 III (2-3 b) | 147 | MW603342 | *Cladosporium cladosporioides* | [KC880082](https://www.ncbi.nlm.nih.gov/nucleotide/528224146?report=genbank&log$=nucltop&blast_rank=1&RID=V49Y5HG9016) | 946 | 99% |
| P3 I (2-1 a) | 148 | MW603343 | Fungal sp. | [KY404936](https://www.ncbi.nlm.nih.gov/nucleotide/1128611656?report=genbank&log$=nucltop&blast_rank=1&RID=VHY1NTY401R) | 950 | 99% |
|  |  |  | *Penicillium viticola* | [NR121209](https://www.ncbi.nlm.nih.gov/nucleotide/662009193?report=genbank&log$=nucltop&blast_rank=2&RID=VHY1NTY401R) | 944 | 99% |
| P3 I (2-1 b) | 149 | MW603344 | *Penicillium herquei* | [KP689192](https://www.ncbi.nlm.nih.gov/nucleotide/755984286?report=genbank&log$=nucltop&blast_rank=1&RID=VHY9786P01R) | 990 | 99% |
| P3 I (2-1 c) | 150 | MW603345 | *Cladosporium ramotenellum* | [KX674646](https://www.ncbi.nlm.nih.gov/nucleotide/KX674646.1?report=genbank&log$=nucltop&blast_rank=1&RID=AYYNFZ0Y014) | 942 | 100% |
| P3 I (2-1 d) | 151 | MW603346 | *Trichoderma harzianum* | [KX346177](https://www.ncbi.nlm.nih.gov/nucleotide/1035343554?report=genbank&log$=nucltop&blast_rank=1&RID=VHYNVT2B01R) | 1070 | 100% |
| P3 I (2-2 a) | 152 | MW603347 | Uncultured fungus | [GQ999239](https://www.ncbi.nlm.nih.gov/nucleotide/299767647?report=genbank&log$=nucltop&blast_rank=1&RID=VKU7MDUZ014) | 979 | 99% |
|  |  |  | *Penicillium wollemiicola* | [KJ174314](https://www.ncbi.nlm.nih.gov/nucleotide/651277165?report=genbank&log$=nucltop&blast_rank=2&RID=VKU7MDUZ014) | 878 | 96% |
| P3 I (2-2 b) | 153 | MW603348 | Uncultured fungus | [GQ999239](https://www.ncbi.nlm.nih.gov/nucleotide/299767647?report=genbank&log$=nucltop&blast_rank=1&RID=VKUS59U2016) | 974 | 99% |
|  |  |  | *Penicillium wollemiicola* | [KJ174314](https://www.ncbi.nlm.nih.gov/nucleotide/651277165?report=genbank&log$=nucltop&blast_rank=2&RID=VKUS59U2016) | 872 | 96% |
| P3 I (2-3 a) | 154 | MW603349 | Fungal sp. | [KU977875](https://www.ncbi.nlm.nih.gov/nucleotide/1152260741?report=genbank&log$=nucltop&blast_rank=1&RID=VKUW1V0N014) | 963 | 99% |
|  |  |  | *Clonostachys byssicola* | [KC806270](https://www.ncbi.nlm.nih.gov/nucleotide/516282546?report=genbank&log$=nucltop&blast_rank=4&RID=VKUW1V0N014) | 963 | 100% |
| P3 I (2-3 b) | 155 | MW603350 | *Cylindrocladium* sp. | [KP972552](https://www.ncbi.nlm.nih.gov/nucleotide/829580571?report=genbank&log$=nucltop&blast_rank=1&RID=VKV0XCZB016) | 959 | 99% |
| P3 II (2-1 b) | 157 | MW603351 | *Penicillium meleagrinum* var. *viridiflavum* | [KT310976](https://www.ncbi.nlm.nih.gov/nucleotide/948273697?report=genbank&log$=nucltop&blast_rank=1&RID=VKV4S309014) | 1016 | 99% |
| P3 II (2-1 c) | 158 | MW603352 | *Cylindrocladium* sp. | [KP972552](https://www.ncbi.nlm.nih.gov/nucleotide/829580571?report=genbank&log$=nucltop&blast_rank=1&RID=VKV88M6A014) | 948 | 99% |
| P3 II (2-1 d) | 159 | MW603353 | *Bionectria* sp. | [GU166503](https://www.ncbi.nlm.nih.gov/nucleotide/312192312?report=genbank&log$=nucltop&blast_rank=1&RID=VKVAX6A6014) | 942 | 99% |
| P3 II (2-1 e) | 160 | MW603354 | *Fusarium decemcellulare* | [KM277988](https://www.ncbi.nlm.nih.gov/nucleotide/748053338?report=genbank&log$=nucltop&blast_rank=1&RID=VKVDVUYJ016) | 953 | 99% |
| P3 II (2-1 f) | 161 | MW603355 | *Penicillium sumatrense* | [KT310939](https://www.ncbi.nlm.nih.gov/nucleotide/948273660?report=genbank&log$=nucltop&blast_rank=1&RID=VKVJFZSG014) | 1394 | 99% |
| P3 II (2-3 a) | 162 | MW603356 | *Penicillium paxilli* | [JN617687](https://www.ncbi.nlm.nih.gov/nucleotide/372123143?report=genbank&log$=nucltop&blast_rank=1&RID=VKVNSUCD016) | 983 | 99% |
| P3 II (2-3 b) | 163 | MW603357 | *Penicillium* sp. | [LC133833](https://www.ncbi.nlm.nih.gov/nucleotide/1040718181?report=genbank&log$=nucltop&blast_rank=1&RID=WH0XH16H01R) | 1009 | 99% |
| P3 II (2-3 c) | 164 | MW603358 | *Hypocrea lixii* | [FJ517550](https://www.ncbi.nlm.nih.gov/nucleotide/219944375?report=genbank&log$=nucltop&blast_rank=1&RID=VKVTP89E014) | 1053 | 99% |
| P3 II (2-2) | 165 | MW603359 | *Pestalotiopsis disseminata* | [KX443705](https://www.ncbi.nlm.nih.gov/nucleotide/KX443705.1?report=genbank&log$=nucltop&blast_rank=1&RID=AYYTRVZV015) | 1044 | 99% |
| P3 III (2-3) | 166 | MW603360 | *Phoma multirostrata* | [KR709058](https://www.ncbi.nlm.nih.gov/nucleotide/967512143?report=genbank&log$=nucltop&blast_rank=1&RID=VKVX9KUR014) | 937 | 100% |
| P4 I (2-2) | 167 | MW603361 | Uncultured fungus | [GQ999256](https://www.ncbi.nlm.nih.gov/nucleotide/GQ999256.1?report=genbank&log$=nucltop&blast_rank=1&RID=AYYZENUR014) | 1258 | 99% |
|  |  |  | *Letendraea helminthicola* | [KU529827](https://www.ncbi.nlm.nih.gov/nucleotide/KU529827.1?report=genbank&log$=nucltop&blast_rank=4&RID=AYYZENUR014) | 1177 | 99% |
| P4 II (2-2) | 168 | MW603362 | Uncultured fungus | [GQ999239](https://www.ncbi.nlm.nih.gov/nucleotide/299767647?report=genbank&log$=nucltop&blast_rank=1&RID=VMJSG6M9014) | 968 | 99% |
|  |  |  | *Penicillium wollemiicola* | [KJ174314](https://www.ncbi.nlm.nih.gov/nucleotide/651277165?report=genbank&log$=nucltop&blast_rank=2&RID=VMJSG6M9014) | 867 | 96% |
| P4 I (2-3 a) | 169 | MW603363 | *Penicillium decaturense* | [HM469399](https://www.ncbi.nlm.nih.gov/nucleotide/326579921?report=genbank&log$=nucltop&blast_rank=1&RID=VMK8N73J01R) | 976 | 99% |
| P4 II (2-3 b) | 170 | MW603364 | *Umbelopsis isabellina* | [LC100011](https://www.ncbi.nlm.nih.gov/nucleotide/LC100011.1?report=genbank&log$=nucltop&blast_rank=1&RID=DX8HKMHG015) | 985 | 98% |
| P4 III (2-2) | 171 | MW603365 | *Hypocrea lixii* | [JX173859](https://www.ncbi.nlm.nih.gov/nucleotide/401878948?report=genbank&log$=nucltop&blast_rank=1&RID=VMKD344Y01R) | 1055 | 99% |
| P4 III (2-3) | 172 | MW603366 | *Bartalinia robillardoides* | [HM802301](https://www.ncbi.nlm.nih.gov/nucleotide/HM802301.1?report=genbank&log$=nucltop&blast_rank=1&RID=60HFHU84013) | 1014 | 99% |
| B1 I (2-1) | 173 | MW603367 | *Trichoderma longibrachiatum* | [HQ833356](https://www.ncbi.nlm.nih.gov/nucleotide/HQ833356.1?report=genbank&log$=nucltop&blast_rank=1&RID=AYZ56J6M014) | 1003 | 99% |
| B1 I (2-3) | 174 | MW603368 | *Fusicolla violacea* | [JN198450](https://www.ncbi.nlm.nih.gov/nucleotide/JN198450.1?report=genbank&log$=nucltop&blast_rank=1&RID=60HN95YK016) | 920 | 99% |
| B1 II (2-1 a) | 175 | MW603369 | Uncultured *Cylindrocladium* | [JX159584](https://www.ncbi.nlm.nih.gov/nucleotide/JX159584.1?report=genbank&log$=nucltop&blast_rank=1&RID=60HYFFX4016) | 708 | 92% |
|  |  |  | *Cylindrocarpon obtusisporum* | [AM419064](https://www.ncbi.nlm.nih.gov/nucleotide/AM419064.1?report=genbank&log$=nucltop&blast_rank=2&RID=60HYFFX4016) | 697 | 92% |
| B1 II (2-1 b) | 176 | MW603370 | *Clonostachys rosea* | [KU350706](https://www.ncbi.nlm.nih.gov/nucleotide/1026943344?report=genbank&log$=nucltop&blast_rank=1&RID=VMKJ2H7P01R) | 944 | 99% |
| B1 II (2-2 a) | 177 | MW603371 | *Hypocrea lixii* | [GU934532](https://www.ncbi.nlm.nih.gov/nucleotide/300676358?report=genbank&log$=nucltop&blast_rank=1&RID=V4A2BWDX016) | 1048 | 99% |
| B1 II (2-2 b) | 178 | MW603372 | Uncultured fungus | [GQ999239](https://www.ncbi.nlm.nih.gov/nucleotide/299767647?report=genbank&log$=nucltop&blast_rank=1&RID=VMKS0G7P01R) | 966 | 99% |
|  |  |  | *Penicillium wollemiicola* | [KJ174314](https://www.ncbi.nlm.nih.gov/nucleotide/651277165?report=genbank&log$=nucltop&blast_rank=2&RID=VMKS0G7P01R) | 865 | 96% |
| B1 II (2-2 c) | 179 | MW603373 | Fungal sp. | [KU977769](https://www.ncbi.nlm.nih.gov/nucleotide/1152260635?report=genbank&log$=nucltop&blast_rank=1&RID=VPEJ5EV8014) | 955 | 99% |
|  |  |  | *Lycoperdon perlatum* | [EU622257](https://www.ncbi.nlm.nih.gov/nucleotide/187472366?report=genbank&log$=nucltop&blast_rank=8&RID=VPEJ5EV8014) | 937 | 99% |
| B1 II (2-3 a) | 180 | MW603374 | *Preussia minimoides* | [AY510423](https://www.ncbi.nlm.nih.gov/nucleotide/46243861?report=genbank&log$=nucltop&blast_rank=1&RID=VCRNH0KP016) | 802 | 97% |
| B1 II (2-3 b) | 181 | MW603375 | *Penicillium* sp. | [JF439498](https://www.ncbi.nlm.nih.gov/nucleotide/328923748?report=genbank&log$=nucltop&blast_rank=1&RID=VPERY0AX014) | 977 | 99% |
| B1 III (2-1) | 182 | MW603376 | Fungal endophyte | [KR080863](https://www.ncbi.nlm.nih.gov/nucleotide/KR080863.1?report=genbank&log$=nucltop&blast_rank=1&RID=DX8T20C6014) | 963 | 98% |
|  |  |  | *Purpureocillium* sp. | [KJ935014](https://www.ncbi.nlm.nih.gov/nucleotide/KJ935014.1?report=genbank&log$=nucltop&blast_rank=2&RID=DX8T20C6014) | 948 | 97% |
| B1 III (2-2) | 183 | MW603377 | *Penicillium viticola* | [NR121209](https://www.ncbi.nlm.nih.gov/nucleotide/662009193?report=genbank&log$=nucltop&blast_rank=1&RID=VPEWAMAM014) | 952 | 99% |
| B1 III (2-3) | 184 | MW603378 | Fungal sp. | [FJ025163](https://www.ncbi.nlm.nih.gov/nucleotide/205277714?report=genbank&log$=nucltop&blast_rank=1&RID=VCRTF032014) | 942 | 99% |
|  |  |  | *Nectria* sp. | [FJ025156](https://www.ncbi.nlm.nih.gov/nucleotide/205277707?report=genbank&log$=nucltop&blast_rank=2&RID=VCRTF032014) | 933 | 99% |
| B2 I (2-1) | 185 | MW603379 | Fungal endophyte | [FJ232907](https://www.ncbi.nlm.nih.gov/nucleotide/209394864?report=genbank&log$=nucltop&blast_rank=1&RID=VCRX89FG014) | 1396 | 99% |
|  |  |  | *Phoma herbarum* | [KP900308](https://www.ncbi.nlm.nih.gov/nucleotide/829098632?report=genbank&log$=nucltop&blast_rank=2&RID=VCRX89FG014) | 911 | 99% |
| B2 I (2-2 a) | 186 | MW603380 | *Mortierella alpina* | [KJ469804](https://www.ncbi.nlm.nih.gov/nucleotide/KJ469804.1?report=genbank&log$=nucltop&blast_rank=1&RID=60J6BUTE013) | 1158 | 99% |
| B2 I (2-2 b) | 187 | MW603381 | *Phoma herbarum* | [KP900244](https://www.ncbi.nlm.nih.gov/nucleotide/829098568?report=genbank&log$=nucltop&blast_rank=1&RID=VCS2GX1R014) | 913 | 99% |
| B2 I (2-3 a) | 188 | MW603382 | *Nectria* sp. | [FJ025156](https://www.ncbi.nlm.nih.gov/nucleotide/205277707?report=genbank&log$=nucltop&blast_rank=1&RID=V1GTRFX7014) | 965 | 99% |
| B2 I (2-3 b) | 189 | MW603383 | Fungal sp. strain | [KT270347](https://www.ncbi.nlm.nih.gov/nucleotide/959586773?report=genbank&log$=nucltop&blast_rank=1&RID=V1H0ASKJ014) | 959 | 99% |
|  |  |  | *Nectria* sp. | [FJ025156](https://www.ncbi.nlm.nih.gov/nucleotide/205277707?report=genbank&log$=nucltop&blast_rank=2&RID=V1H0ASKJ014) | 959 | 99% |
| B2 I (2-3 c) | 190 | MW603384 | Fungal sp. isolate | [KY945036](https://www.ncbi.nlm.nih.gov/nucleotide/1180459680?report=genbank&log$=nucltop&blast_rank=1&RID=V1HA7708014) | 937 | 99% |
|  |  |  | *Cladosporium cladosporioides* | [KY114882](https://www.ncbi.nlm.nih.gov/nucleotide/1148303064?report=genbank&log$=nucltop&blast_rank=2&RID=V1HA7708014) | 937 | 99% |
| B2 II (1-1) | 191 | MW603385 | *Cladosporium* sp. | [JF819132](https://www.ncbi.nlm.nih.gov/nucleotide/JF819132.1?report=genbank&log$=nucltop&blast_rank=1&RID=60JDKKWK016) | 948 | 99% |
| B2 II (1-2 a) | 192 | MW603386 | *Cladosporium* sp. | [KX378909](https://www.ncbi.nlm.nih.gov/nucleotide/1061431352?report=genbank&log$=nucltop&blast_rank=1&RID=VCS67GY2016) | 946 | 99% |
| B2 II (1-2 b) | 193 | MW603387 | *Pestalotiopsis vismiae* | [KP689172](https://www.ncbi.nlm.nih.gov/nucleotide/755984218?report=genbank&log$=nucltop&blast_rank=1&RID=VCS9BCC4014) | 1040 | 99% |
| B2 II (1-3) | 194 | MW603388 | *Bionectria* sp. | [GU166500](https://www.ncbi.nlm.nih.gov/nucleotide/312192310?report=genbank&log$=nucltop&blast_rank=1&RID=VCSD44JP016) | 950 | 99% |
| B2 II (2-1 a) | 195 | MW603389 | *Liberomyces* sp. | [KT336540](https://www.ncbi.nlm.nih.gov/nucleotide/KT336540.1?report=genbank&log$=nucltop&blast_rank=1&RID=60JJPY3D013) | 905 | 99% |
| B2 II (2-1 b) | 196 | MW603390 | *Bionectria* sp. | [GU166500](https://www.ncbi.nlm.nih.gov/nucleotide/312192310?report=genbank&log$=nucltop&blast_rank=1&RID=VCSH9PZ0016) | 955 | 99% |
| B2 II (2-2) | 197 | MW603391 | *Trichoderma viride* | [FJ426393](https://www.ncbi.nlm.nih.gov/nucleotide/FJ426393.1?report=genbank&log$=nucltop&blast_rank=1&RID=60JPZEAS016) | 1048 | 99% |
| B2 II (2-3 a) | 198 | MW603392 | *Acremonium* sp. | [KX034384](https://www.ncbi.nlm.nih.gov/nucleotide/1035335830?report=genbank&log$=nucltop&blast_rank=1&RID=VCSMB1DA014) | 793 | 96% |
| B2 II (2-3 b) | 199 | MW603393 | Fungal sp. | [KY496833](https://www.ncbi.nlm.nih.gov/nucleotide/1204340351?report=genbank&log$=nucltop&blast_rank=1&RID=VCSS6WZU014) | 887 | 99% |
|  |  |  | *Liberomyces* sp. | [KT336540](https://www.ncbi.nlm.nih.gov/nucleotide/969986908?report=genbank&log$=nucltop&blast_rank=2&RID=VCSS6WZU014) | 887 | 99% |
| B2 II (2-3 c) | 200 | MW603394 | *Clonostachys rosea* | [KR909139](https://www.ncbi.nlm.nih.gov/nucleotide/983947093?report=genbank&log$=nucltop&blast_rank=1&RID=VCSXU223014) | 941 | 99% |
| B2 III (1-3 a) | 201 | MW603395 | *Pestalotiopsis vismiae* | [KY810494](https://www.ncbi.nlm.nih.gov/nucleotide/KY810494.1?report=genbank&log$=nucltop&blast_rank=1&RID=60JZK4NU016) | 1040 | 99% |
| B2 III (1-3 b) | 202 | MW603396 | *Cladosporium* sp. | [HQ671188](https://www.ncbi.nlm.nih.gov/nucleotide/317574108?report=genbank&log$=nucltop&blast_rank=1&RID=V4A7RHFH016) | 948 | 99% |
| B2 III (2-1) | 203 | MW603397 | *Clonostachys rosea* | [KU350706](https://www.ncbi.nlm.nih.gov/nucleotide/1026943344?report=genbank&log$=nucltop&blast_rank=1&RID=V1HHVEHA016) | 965 | 99% |
| B2 III (2-2) | 204 | MW603398 | *Pestalotiopsis vismiae* | [KY810494](https://www.ncbi.nlm.nih.gov/nucleotide/1168720208?report=genbank&log$=nucltop&blast_rank=1&RID=V4GC5XKH014) | 1026 | 99% |
| B2 III (2-3 a) | 205 | MW603399 | Ascomycota sp. | [FJ375157](https://www.ncbi.nlm.nih.gov/nucleotide/229597490?report=genbank&log$=nucltop&blast_rank=1&RID=VEJBDT12014) | 800 | 96% |
|  |  |  | *Angustimassarina alni* | [KY548099](https://www.ncbi.nlm.nih.gov/nucleotide/1220005068?report=genbank&log$=nucltop&blast_rank=6&RID=VEJBDT12014) | 719 | 93% |
| B2 III (2-3 b) | 206 | MW603400 | *Clonostachys rosea* | [KU350710](https://www.ncbi.nlm.nih.gov/nucleotide/1026943348?report=genbank&log$=nucltop&blast_rank=1&RID=V1HT156Z014) | 950 | 99% |
| B2 III (2-3 c) | 207 | MW603401 | *Clonostachys rosea* | [KX783354](https://www.ncbi.nlm.nih.gov/nucleotide/1236612992?report=genbank&log$=nucltop&blast_rank=1&RID=V1HZT15K016) | 948 | 99% |
| B2 III (2-3 d) | 208 | MW603402 | *Fusicolla violacea* | [JN198450](https://www.ncbi.nlm.nih.gov/nucleotide/339715435?report=genbank&log$=nucltop&blast_rank=1&RID=V4GM1CXA014) | 931 | 99% |
| B2 III (2-3 e) | 209 | MW603403 | *Xylaria* sp. | [JQ862705](https://www.ncbi.nlm.nih.gov/nucleotide/429888651?report=genbank&log$=nucltop&blast_rank=1&RID=V4GU99T2014) | 983 | 99% |
| B2 III (2-3 f) | 210 | MW603404 | Xylariaceae sp. | [KX722235](https://www.ncbi.nlm.nih.gov/nucleotide/1151248785?report=genbank&log$=nucltop&blast_rank=1&RID=VEJPCTJ1014) | 1694 | 99% |
|  |  |  | *Annulohypoxylon viridistratum* | [KX376325](https://www.ncbi.nlm.nih.gov/nucleotide/1114445796?report=genbank&log$=nucltop&blast_rank=3&RID=VEJPCTJ1014) | 1631 | 99% |
| B3 I (1-1 a) | 211 | MW603405 | *Penicillium herquei* | [MF663569](https://www.ncbi.nlm.nih.gov/nucleotide/1231159550?report=genbank&log$=nucltop&blast_rank=1&RID=VPF0Y272016) | 987 | 99% |
| B3 I (1-1 b) | 212 | MW603406 | *Pestalotiopsis microspora* | [KP689177](https://www.ncbi.nlm.nih.gov/nucleotide/KP689177.1?report=genbank&log$=nucltop&blast_rank=1&RID=60K2Z479016) | 942 | 99% |
| B3 I (1-2 a) | 213 | MW603407 | *Pestalotiopsis cocculi* | [KU324801](https://www.ncbi.nlm.nih.gov/nucleotide/KU324801.1?report=genbank&log$=nucltop&blast_rank=1&RID=60K88P17013) | 1040 | 99% |
| B3 I (1-2 b) | 214 | MW603408 | *Penicillium herquei* | [KP689192](https://www.ncbi.nlm.nih.gov/nucleotide/755984286?report=genbank&log$=nucltop&blast_rank=1&RID=VPFCC58Y016) | 990 | 99% |
| B3 I (1-3 a) | 215 | MW603409 | *Cylindrocladium* sp. | [KP972552](https://www.ncbi.nlm.nih.gov/nucleotide/829580571?report=genbank&log$=nucltop&blast_rank=1&RID=VEK46DYY014) | 948 | 99% |
| B3 I (1-3 b) | 216 | MW603410 | *Penicillium herquei* | [MF663569](https://www.ncbi.nlm.nih.gov/nucleotide/1231159550?report=genbank&log$=nucltop&blast_rank=1&RID=VPFKT1ME014) | 989 | 99% |
| B3 I (2-1 a) | 217 | MW603411 | *Pestalotiopsis microspora* | [KU720061](https://www.ncbi.nlm.nih.gov/nucleotide/KU720061.1?report=genbank&log$=nucltop&blast_rank=1&RID=60M19T71013) | 935 | 99% |
| B3 I (2-1 b) | 218 | MW603412 | *Fusarium solani* | [KX783367](https://www.ncbi.nlm.nih.gov/nucleotide/1236613005?report=genbank&log$=nucltop&blast_rank=1&RID=VEK9WC3U014) | 944 | 99% |
| B3 I (2-2) | 219 | MW603413 | Fungal sp. | [KR012462](https://www.ncbi.nlm.nih.gov/nucleotide/KR012462.1?report=genbank&log$=nucltop&blast_rank=1&RID=60MBZSSN01N) | 1027 | 99% |
|  |  |  | *Pestalotiopsis neglecta* | [JX415485](https://www.ncbi.nlm.nih.gov/nucleotide/JX415485.1?report=genbank&log$=nucltop&blast_rank=3&RID=60MBZSSN01N) | 1020 | 99% |
| B3 II (2-2) | 220 | MW603414 | *Penicillium thomii* | [JN624909](https://www.ncbi.nlm.nih.gov/nucleotide/348161200?report=genbank&log$=nucltop&blast_rank=1&RID=VEKEGY5E014) | 985 | 99% |
| B3 II (2-3) | 221 | MW603415 | *Clonostachys rosea* | [KR909139](https://www.ncbi.nlm.nih.gov/nucleotide/983947093?report=genbank&log$=nucltop&blast_rank=1&RID=VEKMG1RT016) | 953 | 99% |
| B3 III (2-1) | 222 | MW603416 | Uncultured *Trichoderma* | [KJ713222](https://www.ncbi.nlm.nih.gov/nucleotide/645760521?report=genbank&log$=nucltop&blast_rank=1&RID=V1J690UV014) | 1057 | 99% |
| B3 III (2-3) | 223 | MW603417 | *Pestalotiopsis microspora* | [KP689177](https://www.ncbi.nlm.nih.gov/nucleotide/KP689177.1?report=genbank&log$=nucltop&blast_rank=1&RID=60MJRNYJ016) | 942 | 99% |
| B4 I (1-1 b) | 225 | MW603418 | *Cladosporium cladosporioides* | [KC880082](https://www.ncbi.nlm.nih.gov/nucleotide/528224146?report=genbank&log$=nucltop&blast_rank=1&RID=VEKRKCNX016) | 950 | 99% |
| B4 I (1-2 a) | 226 | MW603419 | *Pestalotiopsis microspora* | [KX755256](https://www.ncbi.nlm.nih.gov/nucleotide/KX755256.1?report=genbank&log$=nucltop&blast_rank=1&RID=60MR4K35013) | 1038 | 99% |
| B4 I (1-2 b) | 227 | MW603420 | *Cladosporium* sp. | [HQ671188](https://www.ncbi.nlm.nih.gov/nucleotide/317574108?report=genbank&log$=nucltop&blast_rank=1&RID=V1JDGMZ5016) | 944 | 99% |
| B4 I (1-2 c) | 227b | MW603421 | *Cladosporium tenuissimum* | [KP689183](https://www.ncbi.nlm.nih.gov/nucleotide/755984258?report=genbank&log$=nucltop&blast_rank=1&RID=VPFRV3G4014) | 952 | 99% |
| B4 I (1-3 a) | 228 | MW603422 | *Cladosporium cladosporioides* | [KY290222](https://www.ncbi.nlm.nih.gov/nucleotide/1233055115?report=genbank&log$=nucltop&blast_rank=1&RID=V4GZ2X7E016) | 941 | 99% |
| B4 I (1-3 b) | 229 | MW603423 | *Cladosporium cladosporioides* | [KP689176](https://www.ncbi.nlm.nih.gov/nucleotide/755984232?report=genbank&log$=nucltop&blast_rank=1&RID=V4H69R03014) | 948 | 99% |
| B4 I (2-1) | 230 | MW603424 | *Pestalotiopsis vismiae* | [KY810494](https://www.ncbi.nlm.nih.gov/nucleotide/1168720208?report=genbank&log$=nucltop&blast_rank=1&RID=V4HBWKM7014) | 1022 | 99% |
| B4 I (2-2 a) | 231 | MW603425 | *Xylaria* sp. | [KF928285](https://www.ncbi.nlm.nih.gov/nucleotide/KF928285.1?report=genbank&log$=nucltop&blast_rank=1&RID=60MW4MEB016) | 1000 | 99% |
| B4 I (2-2 b) | 232 | MW603426 | *Trichocladium* sp. | [KF881748](https://www.ncbi.nlm.nih.gov/nucleotide/583842731?report=genbank&log$=nucltop&blast_rank=1&RID=V4HJM186016) | 878 | 98% |
| B4 I (2-2 c) | 233 | MW603427 | Fungal sp. | [KC131406](https://www.ncbi.nlm.nih.gov/nucleotide/533052939?report=genbank&log$=nucltop&blast_rank=1&RID=WH145FZF01R) | 957 | 99% |
|  |  |  | *Xylaria grammica* | [JQ341087](https://www.ncbi.nlm.nih.gov/nucleotide/407378256?report=genbank&log$=nucltop&blast_rank=3&RID=WH145FZF01R) | 955 | 99% |
| B4 I (2-3) | 234 | MW603428 | *Massarina* sp. | [KX788201](https://www.ncbi.nlm.nih.gov/nucleotide/1143348242?report=genbank&log$=nucltop&blast_rank=1&RID=V4HREC9N016) | 876 | 98% |
| B4 II (1-1 a) | 235 | MW603429 | *Cryphonectria* sp. | [KC963928](https://www.ncbi.nlm.nih.gov/nucleotide/528897533?report=genbank&log$=nucltop&blast_rank=1&RID=V4HYAMNJ014) | 1064 | 99% |
| B4 II (1-1 b) | 236 | MW603430 | *Xylaria* sp. | [KF928285](https://www.ncbi.nlm.nih.gov/nucleotide/586829651?report=genbank&log$=nucltop&blast_rank=1&RID=VPFZTYWJ014) | 996 | 99% |
| B4 II (1-1 c) | 237 | MW603431 | *Xylaria* sp. | [JQ862705](https://www.ncbi.nlm.nih.gov/nucleotide/429888651?report=genbank&log$=nucltop&blast_rank=1&RID=VPG4D6FU014) | 1011 | 99% |
| B4 II (1-2 a) | 238 | MW603432 | *Biscogniauxia* sp. | [KP306931](https://www.ncbi.nlm.nih.gov/nucleotide/KP306931.1?report=genbank&log$=nucltop&blast_rank=1&RID=5V3JZ5MB014) | 1175 | 99% |
| B4 II (1-2 b) | 239 | MW603433 | *Podospora intestinacea* | [KM513615](https://www.ncbi.nlm.nih.gov/nucleotide/742522528?report=genbank&log$=nucltop&blast_rank=1&RID=VPG80ER0014) | 819 | 98% |
| B4 II (1-2 c) | 240 | MW603434 | *Fusarium solani* | [KX783350](https://www.ncbi.nlm.nih.gov/nucleotide/1236612988?report=genbank&log$=nucltop&blast_rank=1&RID=VPGCHX5E016) | 948 | 99% |
| B4 II (1-3) | 241 | MW603435 | *Cylindrocladium* sp. | [KP972552](https://www.ncbi.nlm.nih.gov/nucleotide/829580571?report=genbank&log$=nucltop&blast_rank=1&RID=VPH5010V014) | 959 | 99% |
| B4 II (2-1) | 242 | MW603436 | *Cladosporium ramotenellum* | [KP701972](https://www.ncbi.nlm.nih.gov/nucleotide/KP701972.1?report=genbank&log$=nucltop&blast_rank=1&RID=60N0ZYY5013) | 944 | 100% |
| B4 I (2-2) | 243 | MW603437 | *Verticillium* cfr. *aranearum* | [AF108467](https://www.ncbi.nlm.nih.gov/nucleotide/4836219?report=genbank&log$=nucltop&blast_rank=1&RID=VPHCZ1SD014) | 1024 | 99% |
| B4 II (2-3) | 244 | MW603438 | *Bionectria ochroleuca* | [FJ238113](https://www.ncbi.nlm.nih.gov/nucleotide/209967460?report=genbank&log$=nucltop&blast_rank=1&RID=V1JHNK0G016) | 955 | 99% |
| B4 II (1-1 d) | 245 | MW603439 | Ascomycete sp. | [AJ972831](https://www.ncbi.nlm.nih.gov/nucleotide/66990780?report=genbank&log$=nucltop&blast_rank=1&RID=V4J61Z88014) | 773 | 91% |
|  |  |  | *Exophiala sideris* | [HQ452316](https://www.ncbi.nlm.nih.gov/nucleotide/330847346?report=genbank&log$=nucltop&blast_rank=3&RID=V4J61Z88014) | 669 | 88% |
| B4 II (1-1 e) | 246 | MW603440 | Chaetothyriales sp. | [KF614883](https://www.ncbi.nlm.nih.gov/nucleotide/KF614883.1?report=genbank&log$=nucltop&blast_rank=1&RID=60N5HBR101N) | 667 | 86% |
|  |  |  | *Capronia munkii* | [NR121263](https://www.ncbi.nlm.nih.gov/nucleotide/NR_121263.1?report=genbank&log$=nucltop&blast_rank=3&RID=60N5HBR101N) | 652 | 86% |
| B4 II (1-1 f) | 247 | MW603441 | Chaetothyriales sp. | [KF614883](https://www.ncbi.nlm.nih.gov/nucleotide/KF614883.1?report=genbank&log$=nucltop&blast_rank=1&RID=60NBY57V013) | 651 | 86% |
|  |  |  | *Capronia munkii* | [NR121263](https://www.ncbi.nlm.nih.gov/nucleotide/NR_121263.1?report=genbank&log$=nucltop&blast_rank=3&RID=60NBY57V013) | 636 | 86% |
| B4 III (1-1) | 248 | MW603442 | *Cladosporium ramotenellum* | [KX674646](https://www.ncbi.nlm.nih.gov/nucleotide/1153124382?report=genbank&log$=nucltop&blast_rank=1&RID=V1JPAS73014) | 939 | 99% |
| B4 III (1-2) | 249 | MW603443 | *Bionectria* sp. | [GU166500](https://www.ncbi.nlm.nih.gov/nucleotide/312192310?report=genbank&log$=nucltop&blast_rank=1&RID=VPJNJTFA014) | 955 | 99% |
| B4 III (1-3) | 250 | MW603444 | *Aspergillus niveus* | [JN246075](https://www.ncbi.nlm.nih.gov/nucleotide/353559095?report=genbank&log$=nucltop&blast_rank=1&RID=VPJVXRXZ016) | 924 | 98% |
| B4 III (2-1) | 251 | MW603445 | *Alternaria* sp. | [KF367473](https://www.ncbi.nlm.nih.gov/nucleotide/530341686?report=genbank&log$=nucltop&blast_rank=1&RID=VPK21142014) | 1018 | 99% |
| B4 III (2-2) | 252 | MW603446 | *Engyodontium* sp. | [KF768338](https://www.ncbi.nlm.nih.gov/nucleotide/574604000?report=genbank&log$=nucltop&blast_rank=1&RID=WH1KY9CW01R) | 992 | 99% |
| B4 III (2-3 a) | 253 | MW603447 | *Exophiala moniliae* | [HE605213](https://www.ncbi.nlm.nih.gov/nucleotide/380350103?report=genbank&log$=nucltop&blast_rank=1&RID=V4JF3MNK016) | 1007 | 97% |
| P3 I (2-1) | 255 | MW603448 | *Cladosporium* sp. | [AF261660](https://www.ncbi.nlm.nih.gov/nucleotide/AF261660.1?report=genbank&log$=nucltop&blast_rank=1&RID=60NK3FAS01N) | 931 | 100% |
| N3 (2-1) | 256 | MW603449 | *Clonostachys rosea* | [KJ540101](https://www.ncbi.nlm.nih.gov/nucleotide/613845931?report=genbank&log$=nucltop&blast_rank=1&RID=VPK5VF1B014) | 957 | 99% |
| N4 III (2-2 2-5) | 257 | MW603450 | Fungal sp. | [FJ025163](https://www.ncbi.nlm.nih.gov/nucleotide/205277714?report=genbank&log$=nucltop&blast_rank=1&RID=VEKX25X4014) | 957 | 99% |
|  |  |  | *Clonostachys rosea* | [KJ540089](https://www.ncbi.nlm.nih.gov/nucleotide/613845893?report=genbank&log$=nucltop&blast_rank=2&RID=VEKX25X4014) | 957 | 99% |
| N4 I (2-3) | 258 | MW603451 | Dothideomycetes sp. | [AB986429](https://www.ncbi.nlm.nih.gov/nucleotide/752503391?report=genbank&log$=nucltop&blast_rank=1&RID=V1JX6692016) | 883 | 97% |

**Supplementary Table S2**. Mantel tests between the distance matrices of the geographic coordinates and the fungal community composition.

|  |  |  |  | |  |  |  |  |  |  |
| --- | --- | --- | --- | --- | --- | --- | --- | --- | --- | --- |
|  | ITS all | | | | | ITS orchid | | | | |
|  | orchids | | bark | | | orchids | | | bark | |
|  | R | P value | R | P value | | R | | P value | R | P value |
| low | -0.169 | 0.787 | 0.084 | 0.269 | | 0.248 | | 0.139 | 0.113 | 0.274 |
| medium | -0.170 | 0.881 | -0.088 | 0.750 | | -0.065 | | 0.600 | -0.087 | 0.686 |
| high | 0.004 | 0.500 | 0.084 | 0.272 | | 0.336 | | **0.038** | 0.113 | 0.284 |
